# Biochar–vermicompost–inorganic N/P₂O₅ integration improves maize yield and soil chemical properties in acidic soils

**DOI:** 10.1038/s41598-026-56112-5

**Published:** 2026-06-29

**Authors:** Habtamu Tadele, Tesfaye Feyisa, Lewoye Tsegaye, Sintayehu Musie

**Affiliations:** 1 Department of Natural Resource Management, College of Agriculture and Environmental Sciences, Bahir Dar University, Bahir Dar, Ethiopia; 2 Department of Natural Resource Management, College of Agriculture and Natural Resources, Debre Markos University, Burie Campus, PO. Box. 269, Burie, Ethiopia; 3 Soil and Water Research Directorate, Amhara Agricultural Research Institute, Bahir Dar, Ethiopia; 4 Crop and Horticulture Science, Department of Horticulture, Debre Markos University, Debre Markos, Ethiopia

**Keywords:** Context-specific, Limitations, Soil chemical quality, Integrated soil fertility management, Vermicompost, Ecology, Ecology, Environmental sciences, Plant sciences

## Abstract

Improving soil chemical quality while enhancing maize yield is critical for sustainable crop production in acidic soils. We conducted a two-year field experiment on acidic Nitisols in the Burie district, Ethiopia, to evaluate the combined effects of maize cob biochar (BC), vermicompost (VC), and inorganic N/P₂O₅ rates on soil chemical properties and maize productivity. The experiment used a randomized complete block design with a 3 × 3 × 3 factorial arrangement, testing three levels of inorganic N/P₂O₅ (0/0, 120/69, 240/138 kg ha^−1^), BC (0, 4, 8 t ha^−1^), and VC (0, 5.02, 10.04 t ha^−1^). Grain yield was recorded annually, and soil samples were collected before and after the experiment to assess changes in chemical properties. Data were analyzed using factorial ANOVA, and treatment means were separated using Tukey’s HSD at 5% significance. Economic feasibility was evaluated via partial budget analysis. Under the conditions of this experiment, integrated application of BC, VC, and reduced N/P₂O₅, alongside lime, significantly improved soil pH (4.87 to 5.82), soil organic carbon, total nitrogen, and available phosphorus, while reducing exchangeable acidity, hydrogen, and aluminum. Maize grain yield increased markedly, with the highest yield (12.13 t ha^−1^) observed under 120/69 kg N/P₂O₅ + 8 t BC + 10.04 t VC, a 175.77% increase over the control (4.40 t ha^−1^). The combination 120/69 kg N/P₂O₅ ha^−1^ + 4 t BC ha^−1^ + 5 t VC ha^−1^ achieved consistently high yields (12.09 t ha^−1^) and the greatest net benefit (1,861 USD ha^−1^; 289,124 Ethiopian Birr ha^−1^) with a marginal rate of return of 149.1%. Yield was strongly positively correlated with improved soil chemical properties. Among predictive models, Linear Support Vector Machine (LSVM) provided the highest accuracy (R^2^ = 0.923) for estimating maize yield under integrated nutrient management. These results indicate that integrating BC and VC with reduced inorganic N/P₂O₅, in combination with lime, can enhance soil chemical quality and maize productivity in acidic Nitisols. These findings suggest potential for sustainable intensification in the study area and similar agro-ecologies, although multi-location and longer-term validation is needed to confirm broader applicability. Further research is needed across diverse environments and longer timeframes.

**Supplementary Information:**

The online version contains supplementary material available at 10.1038/s41598-026-56112-5.

## Introduction

Maize (*Zea mays* L.) is a major staple crop in Ethiopia and a key source of food security and income for smallholder farmers. However, its productivity in the highlands of Northwest Ethiopia, including the Burie district, remains far below its potential due to declining soil fertility, soil acidity, and continuous nutrient depletion. In these systems, Nitisols dominate and are characterized by low soil organic matter (SOM), nitrogen (N) and phosphorus (P) deficiencies, and aluminum (Al) toxicity, all of which restrict crop growth and nutrient availability^1,2^. In addition, limited biomass returns to the soil and continuous cultivation have accelerated soil degradation across the Ethiopian highlands^3^. The challenge is further compounded by the fact that reliance on either inorganic or organic fertilizers alone cannot sustain crop productivity over time^4^. In acidic soils, high concentrations of Al and Fe exacerbate P fixation and reduce nutrient availability^5^. Liming has been widely used to mitigate soil acidity by reducing the solubility of toxic elements such as Al and Mn, thereby improving nutrient availability^6^.

In this context, integrated soil fertility management (ISFM), which combines organic and inorganic inputs, has emerged as a promising strategy to restore soil fertility and enhance crop productivity. The integration of organic amendments with mineral fertilizers improves nutrient availability, increases soil buffering capacity, enhances soil organic carbon, and promotes better moisture retention^7,8^. In Ethiopia, such integrated approaches have consistently outperformed sole applications, with significant improvements in both soil properties and crop yields^9,10^. Among organic amendments, BC and VC are particularly important due to their complementary roles in improving soil pH, cation exchange capacity, nutrient supply, and microbial activity^11–13^.

Integrated nutrient management studies involving organic amendments have been widely reported in tropical soils^12–14^, despite these advances, important research gaps remain. First, most previous studies have examined binary combinations (e.g., fertilizer + biochar or fertilizer + compost) rather than three-way interactions among inorganic fertilizers, biochar, and vermicompost. Second, the specific effects of combining biochar (produced from maize cobs) with nutrient-rich vermicompost on soil chemical property dynamics in very strongly acidic Nitisols have not been systematically quantified. Third, few studies have established quantitative correlations between specific soil property improvements and yield gains under integrated management. Specifically, the mechanisms by which combined applications alleviate Al toxicity (through pH increase vs. organic complexation), enhance P availability (through reduced fixation vs. direct supply), and improve N use efficiency (through reduced leaching vs. enhanced mineralization) in acidic Nitisols remain poorly understood. This study addresses these mechanistic questions through comprehensive soil analysis and correlation with crop response.

Under Ethiopian conditions, there is a lack of comprehensive field-based studies that evaluate the combined and interactive effects of BC, VC, and inorganic N/P₂O₅ nutrients on both soil chemical quality and maize grain yield. Although Tadele et al.^15^,Tadele et al.^16^,Tadele et al.^17^, reported crop phenology, growth, and sustainability indices at the same site, the dynamics of soil chemical properties and their linkage with yield have not been fully explored. To our knowledge, this is the first study in Ethiopian Nitisols to comprehensively examine three-way interactions among these specific amendments while simultaneously quantifying both soil chemical property changes and their direct correlations with maize yield.

Therefore, this study aims to address these gaps by providing an integrated assessment of soil chemical properties (i.e. acidity and fertility) and maize grain yield under ISFM practices in acidic Nitisols. Specifically, the objectives were to: (i) quantify the synergistic effects of the three-way interaction (N/P₂O₅ × BC × VC) on soil chemical quality and yield; (ii) establish relationships between soil property improvements and yield response; (iii) evaluate whether reduced rates (50%) of inorganic N/P₂O₅ combined with organic amendments can achieve comparable or higher yields than full recommended rates; and (iv) assess and compare the predictive performance of different models, including Multiple Linear Regression Model (MLRM), Linear Support Vector Machine (LSVM), Bayesian Network Model (BNM), and Neural Network Model (NNM).

Accordingly, the study tested the hypotheses that (H_o_) Integrating BC and VC with reduced rates of inorganic N/P₂O₅ does not significantly affect soil chemical properties (pH, exchangeable acidity, available P, SOC, TN) compared with inorganic N/P_2_O_5_ alone under acidic Nitisols, (H_a1_) Integrating BC and VC with reduced rates of inorganic N/P₂O₅ significantly improves soil chemical properties compared with inorganic fertilizers alone, (H_a2_) Maize grain yield under combined BC, VC, and reduced inorganic N/P₂O₅ rates does not differ from yield under the full recommended rate of inorganic N/P_2_O_5_, (H_a3_): Maize grain yield under combined BC, VC, and reduced inorganic N/P₂O₅ rates is comparable to or greater than yield under the full recommended rate, (H_a4_) : No significant correlation exists between soil chemical properties and maize grain yield across treatments, (H_a5_): Improvements in soil chemical properties resulting from integrated treatments are positively and significantly correlated with maize grain yield. Additionally, (H_a6_) advanced machine learning models will outperform conventional regression approaches in predicting maize grain yield, as indicated by superior model performance metrics (e.g., higher R^2^ and lower RMSE and MAE). This study provides ideal evidence on the three-way interaction effects under the conditions of this experiment and offers practical insights for sustainable soil fertility management in the Burie district.

## Materials and methods

### Experimental site

**Fig. 1:**
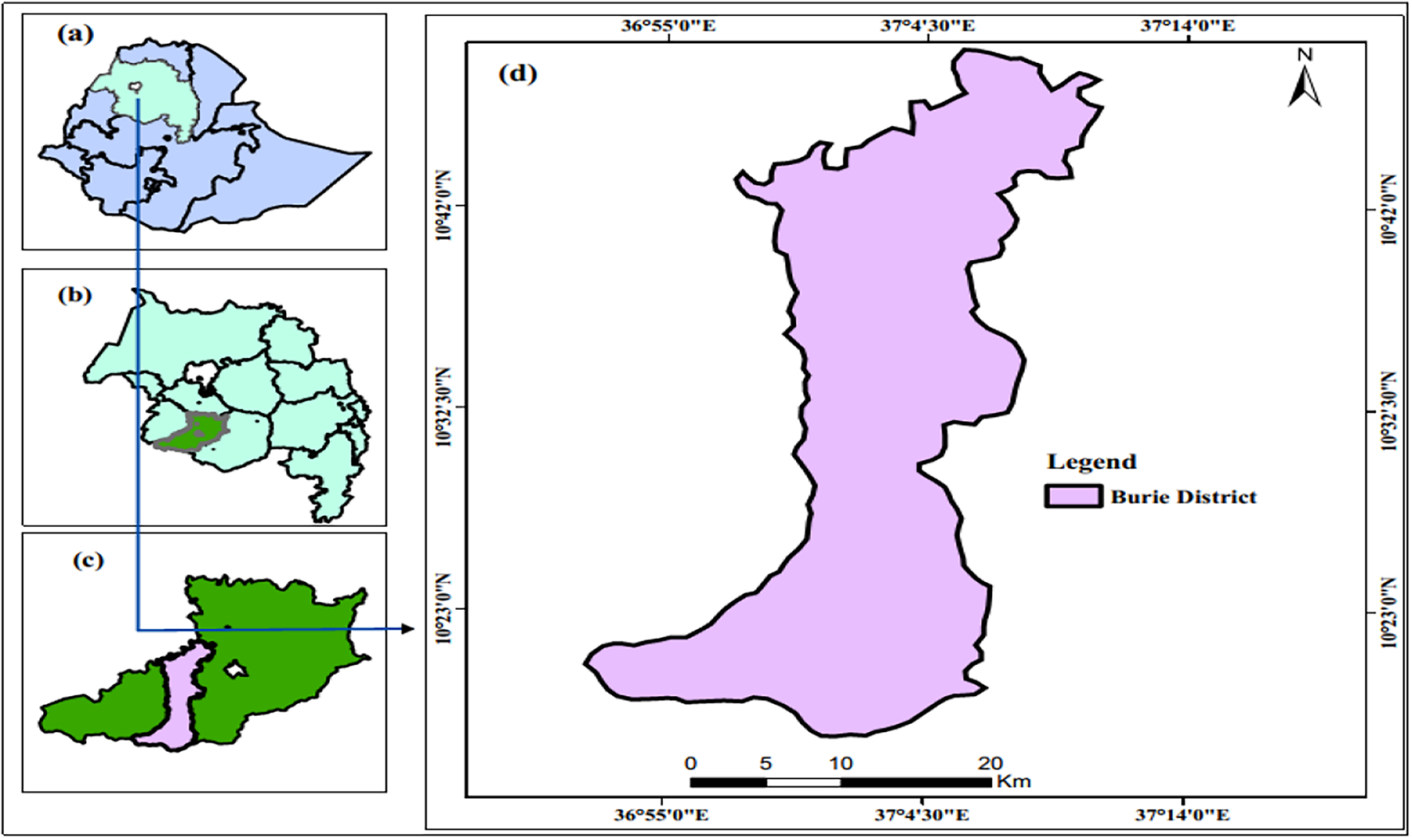
Study area map showing: (**a**) Amhara Region within Ethiopia, (**b**) West Gojjam Zone within the Amhara Region, (**c**) Burie district within West Gojjam Zone, and (**d**) the specific study site in Burie District (Habtamu Tadele, ArcGIS, 2023).

**Fig. 2:**
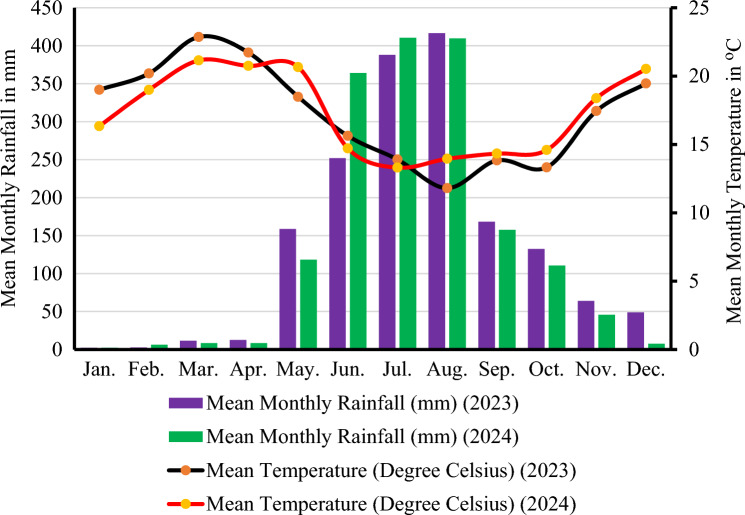
Mean monthly rainfall (mm) and mean monthly temperatures (°C) of the study area recorded from 2023/24–2024/25.

A field study was conducted over two consecutive main cropping seasons (2023/24–2024/25) at the Debre Markos University, Burie Campus research farm site in the Burie district (latitude 10°23′0’’–10°42′0’’ N, longitude 37°14′0’’–37°55′0’’ E; altitude 2146–2148 m.a.s.l.). The experimental plot was specifically located at 10°42′38.99″ N, 37°4′44.36″ E (Fig. 1). The main cropping seasons align with the region’s rainfall periods, providing sufficient soil moisture for maize growth. The climate is sub-humid, with unimodal rainfall totaling 1654 mm annually (2023–2025 mean), ranging from 2.5 mm in January to 416 mm in August. Mean monthly air temperatures range from 14.5 °C (July–August) to 20.2 °C (March–April), with an annual mean of 17.3 °C (Fig. 2). These data were obtained from the Amhara Regional State Meteorological Agency (unpublished data, 2023–2025) and represent the most complete records available for the study period. Although long-term normals would be preferable, the two-year meteorological data provide important context for the experimental conditions during the study. Prior to the experiment, the field had been cultivated with maize and faba bean, potentially influencing baseline soil fertility. Initial soil sampling was conducted to determine background nutrient levels. Faba bean was previously managed under conventional practices typical of the area, including recommended spacing, standard basal fertilizer application, and manual weeding.

### Experimental materials

Bako hybrid maize (BH-661), obtained from the Certified Seed Multiplication Center in Burie district, was used as the test crop at a seed rate of 42.61 kg ha^−1^, following the agronomic recommendations of the Amhara Regional State Agricultural Extension Agronomy Package (unpublished manual, 2022) and^18^. This single-cross hybrid performs well at altitudes of 1600–2200 m above sea level, with research yields ranging from 9.5 to 12 t ha^−1^ and farmer yields ranging from 6.5 to 8.5 t ha^−1^ (Amhara Regional State Agricultural Extension Agronomy Package, unpublished manual, 2022).

### Experimental design, treatments, and replication

The experiment was conducted in a randomized complete block design (RCBD) with 27 treatments (3 × 3 × 3 factorial) and three replications, with each plot being 3 × 2.75 m (8.25 m^2^ gross; 3.63 m^2^ net harvest area). The experimental area covers 922.25 m^2^ (29.75 × 31 m). The factors included inorganic N/P_2_O_5_ levels: 0/0, 120/69, 240/138 kg N/P₂O₅ ha^−1^, applied as urea (46% N) and blended NPSB (nitrogen, phosphorus, sulfur and boron) fertilizer (18.9% N, 36.1% P₂O₅, 6.95% S, 0.1% B); BC levels: 0, 4, 8 t ha^−1^; and VC levels: 0, 5.02, 10.04 t ha^−1^. The confounding effects of sulfur and boron supplied through the blended fertilizer were not separately considered, similar to the approach used for lime. Detailed treatments are provided in Supplementary Table S1.

The fully recommended N/P_2_O_5_ level for the study (240 kg N ha^−1^, 138 kg P₂O₅ ha^−1^) was used as recommended by Worku et al.^19^ for this variety and plant population, and the unpublished Amhara Regional State Agronomy Manual (2022). The inorganic N/P₂O₅ rates were selected based on: (i) the full recommended rate for maize in the study area (240 kg N ha^−1^ and 138 kg P₂O₅ ha^−1^) described by Worku et al.^18^ in the Amhara highlands,(ii) 50% of the recommended rate (120/69 kg N/P₂O₅ ha^−1^) to test the hypothesis that organic amendments can partially substitute for inorganic fertilizers; and (iii) a control (0 kg ha^−1^) to establish baseline response. The recommended rate of 240 kg N ha^−1^ aligns with the Amhara Regional State extension package for high-yielding maize hybrids in areas with reliable rainfall^18^, while the P rate accounts for the severe P fixation characteristic of acidic Nitisols^1^. Phosphorus omission treatments were excluded because severe P deficiency is widespread in the Amhara region^14^, and complete omission would not reflect realistic farmer practices.

Biochar rates of 4 and 8 t ha^−1^ were selected based on: (i) a review of tropical soil studies showing significant pH and nutrient improvements within this range^13,19^; (ii) practical feasibility for smallholder farmers considering biomass availability (maize cob production in the district averages 3–5 t ha^−1^ annually); (iii) preliminary on-station trials in Ethiopia indicating 4–8 t ha^−1^ as optimal for acidic soils^20,21^; (iv) economic considerations balancing amendment cost against expected yield benefits; and (v) the need to test whether higher rates (8 t ha^−1^) provide additional benefits beyond moderate application (4 t ha^−1^).

Dejen agricultural lime (CaCO₃) was applied uniformly to all plots, including the control, at sowing.

The partial application of lime (25% of total lime requirement for row application method) aimed to reduce the confounding effects of severe soil acidity while enabling evaluation of treatment differences under moderately corrected conditions (unpublished manual, 2022). By applying lime uniformly, a baseline soil acidity level was established, allowing the specific effects of BC, VC, and their interactions with inorganic N/P₂O₅ nutrients to be isolated. Therefore, all treatment effects reported in this study reflect the combined influence of lime plus the respective amendments, rather than the amendments alone. The lime requirement (LR) was calculated as described by Kamprath^22^ and Tadele et al.^16^:

1 $$LR(tha^{( - 1)} ) = lf \times Exc.Ac$$

LR, lime requirement; Exc. Ac, the initial exchangeable acidity of the soil (H^+^ and Al^3+^); lf, the lime factor, which is 1.5 times the initial exchangeable acidity (cmol_(c)_ kg^-1^) will be enough to reduce the acidity saturation to at most 15%, which will be considered to be a threshold below which most crops are not affected by acidity. The nutrient sources, application rates, sowing dates, and urea split schedules are summarized in .

### Field layout and crop establishment

The experimental area (922.25 m^2^; 29.75 m × 31 m) was arranged with 0.5 m pathways between adjacent plots and 1 m pathways between replications to facilitate field operations and minimize cross-contamination. Each plot (3 m × 2.75 m = 8.25 m^2^ gross area) consisted of five rows spaced 0.55 m apart, with plants within rows spaced 0.20 m apart, giving 15 plants per row and a total population of 90,909 plants ha^−1^. Border effects were minimized by: (i) harvesting only the central three rows (excluding two outer rows on each side); (ii) excluding the first and last plants in each harvested row; and (iii) maintaining the net harvest area as 1.65 m × 2.20 m = 3.63 m^2^. This approach follows standard practice for maize field trials^16,18^ and ensures that yield estimates are based on competition-free plants. While plots of 8.25 m^2^ are smaller than typical farmer fields, they are standard for controlled agronomic trials where multiple treatments must be accommodated within a limited area^14,17^. Permanent plots were maintained over two years. The soil was plowed three times: initial plowing with a disc plow, secondary plowing with an oxen-driven plow (*Maresha*), and final levelling by hand to avoid cross-contamination. Bunds and ditches (50 cm deep) surrounded the plots for drainage.

Sowing was conducted manually at 7 cm: two seeds per hill were thinned to one plant after two weeks. Nitrogen was applied in split parts: 28.83% at planting, and the remainder was applied in two stages (50 cm height and tasseling) (Table 1). Phosphorus was applied at planting according to the treatment. BC and VC were applied in rows at sowing, with VC rates adjusted on the basis of nitrogen content^14,23^. Two rounds of hand weeding (25 and 45 days after planting, DAP) ensured minimal nutrient competition and controlled plant growth. Other agronomic practices followed agronomics recommendations^21,24^.

**Table 1:** Sources of inorganic and organic nutrients, application dates, and sowing times.

Sources of nutrients	Amount (kg ha^−1^)	Nutrients (kg ha^−1^)		Application time
		N	P_2_O_5_	
Blended NPSB	366.05	69.2	138	At sowing
Urea (CO (NH_2_)_2_	371.34	170.8	––	
Year	2023/24	2024/25		
Sowing date	June 8	May 28	––	
1^st^ urea split	July 18	July 6	––	At knee height stage (40 DAS)
2^nd^ urea split	September 6	August 20	––	At tasseling initiation stage

DAS, days after sowing.

### Biochar and VC production, analysis and application

The biochar (BC) used in this study was produced from air-dried maize cobs (210 kg) through pit pyrolysis using a pit measuring 2 × 1.5 × 1 m, conducted on-site during sunny daytime conditions^15^. The biochar was produced using the traditional pit method; therefore, a specific pyrolysis temperature was not directly controlled or measured. The biomass was ignited and allowed to smolder until visible smoke ceased, yielding 42.35% biochar. After cooling, the biochar was crushed, sieved to < 5 mm, and stored in sealed bags until use. The key chemical properties of the biochar were: pH (9.88), total carbon (64.62%), total nitrogen (1.11%), and a C:N ratio of 58:1. Biochar was applied at rates of 0, 4, and 8 t ha^−1^ (equivalent to 0, 3.3, and 6.6 kg per plot), respectively. Prior to application, it was moistened with water to reduce dust, following^25^ and applied twice per cropping season. Fine particles (< 0.5 mm) were used for OC and TN analysis, and < 2 mm for pH and available P measurement^26^. The observed biochar properties are consistent with pit pyrolysis conditions at low to moderate temperatures, as described by Adekanye et al.^27^ and Rahmat et al.^28^. For pH measurement, 1 g of BC was mixed with 20 mL of distilled water (1:20 ratio) following^26^. Yield was calculated as described by Eq. 2, while ash content, CEC, and EC were not determined.

2 $$Biochar yield \left( \% \right) = \frac{{Weight of biochar \left( {kg} \right)}}{{weight of maize cob \left( {kg} \right)}} \times 100\%$$

Vermicompost was prepared from local organic materials and analyzed for total nitrogen before application. It was applied at 0, 5.02, and 10.04 t ha^−1^, based on the TN content in each year, while the full recommended rates were 11.12 t ha^−1^ (2023) and 8.96 t ha^−1^ (2024)^15^. Vermicompost was applied in rows at planting alongside biochar and inorganic fertilizers. An earthworm bin (2 × 0.6 × 0.5 m) was layered with chopped *Vernonia auriculifera* (10 cm), 20 cm old cow dung, and 10 cm fertile topsoil, repeated to fill the bin. The bin was watered every 2 days and turned daily for 14 days to maintain 70–80% moisture. After 15 days of pre-composting, ***Eisenia fetida***, an *epigeic* earthworm species were added at a 2:1 worm-to-feedstock ratio. This ratio was selected based on established vermicomposting practices, which suggest a sufficient worm biomass relative to feedstock ensures efficient decomposition, optimal nutrient mineralization, and avoidance of overstocking that can stress the worms^12,29^. The bin was covered with green leaves for 60 days to maintain humidity and prevent pests^21^. Vermicompost was harvested via light separation, sieved to remove roots and impurities, and stored in the shade. Moisture content was determined by oven-drying at 105 °C for 24 h. Fresh VC was applied at seeding, with rates calculated on a dry-weight basis.

Nitrogen equivalence of VC (NEvc): During the 2023/24 and 2024/25 cropping seasons, the TN content of VC was measured prior to application. The total nitrogen equivalence (NE_tot_) of vermicompost was.

The nitrogen equivalence of VC (NEvc) was calculated using Eq. 3 as described by Tadele et al.^16^:

3 $$NEq_{vc} = 10 \times TN_{vc} \left( \% \right)$$

Abbreviations: NEq_vc_, the nitrogen equivalence VC t^−1^; TN is the total nitrogen (%) per 100 kg of VC; 10 is the conversion factor used to scale the nitrogen content from 100 kg to 1 tonne.

This nitrogen-based approach ensures that comparisons between organic and inorganic treatments are grounded in equivalent nitrogen supply potential, while acknowledging that actual availability differs between organic and mineral sources^23^. Analysis of VC properties: selected parameters of VC were determined via dried samples, which were ground to pass through a 2 mm sieve as described by Pisa and Wuta^30^. The pH of VC was determined from a suspension of 1:10 VC:H_2_O as described by Ndegwa and Thompson^31^. The total OC content of VC was estimated via the wet combustion procedure of Walkley and Black^32^. The OM (%) of VC was obtained by multiplying the total OC% by 1.8^33^. The TN content of VC was determined via the wet-oxidation procedure of the Kjeldahl method^32^. Total P was extracted using a mixture of concentrated H_2_SO_4_, Se powder, salicylic acid (C_7_H_6_O_3_), and H_2_O_2_^34^.

### Field management

Weeding, hoeing, and pest/disease management were conducted according to standard agronomic practices^14,24,35^. To avoid border effects, only the central area of each plot with two plants in each row was harvested. All the treatments received identical lime and field management practices except for the applied amendments (Table 1).

### Soil sampling and analysis

The composite sampling approach (10 cores mixed) follows standard protocols for initial site characterization^36^ and provides a reliable estimate of mean soil properties across the experimental area. The relatively uniform topography and consistent cropping history (previous maize-faba bean rotation) suggest that spatial variability was moderate. Initial soil sampling was conducted before the start of the experiment (February 10, 2023) at a depth of 0–20 cm using a manual soil auger. Ten random samples were collected in a zigzag pattern across the experimental field and composited to obtain one representative sample for analysis of baseline soil physicochemical properties. Similarly, 10 undisturbed core samples were collected for the determination of soil physical properties.

For soil sampling after harvesting from each plot, an auger was used to sample five randomly selected locations per plot. These five subsample soils were combined into one composite soil sample per plot to investigate the soil properties of each treatment. A manual soil auger with a knife-shaped tip (20 cm depth) and a total length of approximately 140 cm was used to collect the soil samples. After harvest, soil samples were also taken from each plot at 0–20 cm depth on February 20^th^, 2025. Hence, with the exception of the soil texture, all the other soil chemical properties were analyzed following the standards. The composite samples were air dried, prepared, and homogenized to analyze the chemical characteristics of the soil, such as Exc. Al^3+^, Exc. H^+^, pH, soil OC, SOM, soil TN, Exc. Ac, and soil avP. The composite surface soil samples were labeled properly. The labels were placed both inside and outside the plastic bags and were transported to the soil laboratory of Bahir Dar University Soil Physics and Plant Analysis.

### Soil sample collection, preparation, and analysis of soil chemical properties

The composite soil samples were air-dried, mixed well, passed through a 2 mm sieve for the analysis of soil pH, Exc. Ac, and avP, and passed through a 0.5 mm sieve for the analysis of SOC and TN^15^. Finally, the soil samples were analyzed following standard procedures^36^. The soil pH was measured at a 1:2.5 soil: water ratio via a pH model (pH 100) pH meter^37^. The total N content was determined via micro-Kjeldahl digestion, distillation, and titration procedures^38^. The soil OC content was determined via the Walkley and Black wet digestion method^32^. The soil OM content was calculated by multiplying the percentage of organic carbon by a factor of 1.724^32^. Soil avP was extracted via the Mehlich-III multi-nutrient extraction method^39^. Soil Exc. Al and Exc. H was determined via the extraction method (1 N KCl extraction). Exchangeable acidity was achieved by weighing 5 g of soil sample, which was leached with potassium chloride (K^+^ ions replace Exc. H^+^ and Al^3+^ held against permanent negative charges of the exchange complex). It was determined by titrating with NaOH.

### Data collected and analysis

Soil samples were collected after harvest from each treatment. Grain yield data were collected in both growing seasons. Grain yield data were collected after sun-drying. When the cobs were threshed by hand, the yield per plot was recorded. Grain yield was determined by weighing grains shelled from ears collected from the central three rows of each plot and expressed on a kg ha^−1^ basis via Eq. 3^16,24^:

4 $$Grain yield (kg^{ - 1} ) = \left( {\frac{{Grain yield (kg plot^{ - 1} )}}{{net area harvested \left( {1.65 m \times 2.5 m} \right)}}} \right) \times 10, 000$$

The threshed grains were air-dried and adjusted to 12.5% moisture content by weight since the standard moisture content of maize and other cereals in Ethiopia is 12.5%^24,40^. Moreover, grain yield was adjusted on the basis of the adjusted moisture content (Eq. (4). The weighed grain yield was then converted into weight per unit area and expressed as t ha^−1^.

5 $${\text{Adjusted grain yield}} = \frac{{Grain yield (kg ha^{ - 1} ) \times \left( {100 - MC} \right)}}{{\left( {100 - 12.5} \right)}}$$

Abbreviations: MC is measured moisture content (%) in the grain and 12.5 is the designated moisture content (%).

Grain moisture content at harvest was measured immediately after threshing using a calibrated moisture meter for each plot. The mean moisture content across treatments was 15.59% in 2023 and 14.68% in 2024. All grain yields were adjusted to 12.5% moisture content (the Ethiopian standard for maize^24,41–43^ using Eq. 5. The reported yields therefore represent dry-weight equivalents at standard moisture, not fresh harvest weights.

### Economic and statistical analysis

#### Economic analysis

A partial budget analysis was conducted using mean adjusted grain and straw yields. For economic analysis of the market price of maize grain yield, the costs of inputs and labor are presented in Table 2. The product of the maize yield for each treatment-year combination was used to calculate the revenue (R). The benefits were calculated as the difference between revenues and expenditures. The net benefit and marginal rate of return (MRR) were computed on the basis of^44^ to determine economic viability via Eq. 6. A 10% yield adjustment was applied to account for farmer-level realities and postharvest losses and reflect real-world variability, as demonstrated by^24,45^. Since our study was conducted at the research farm site.

6 $$MRR \left( \% \right) = \frac{{NB_{b } - NB_{a } }}{{TVC_{b } - TVC_{a } }} \times 100$$

**Table 2:** Market prices of inputs and labor costs for economic analysis (ETB; 1 USD = 55.09 ETB, mean exchange rate).

Sn	Items	Year		Mean (ETB)
		2023/24	2024/25	
1	Urea (100 kg^−1^)	8589.98	4060.00	6324.99
2	NPSB (100 kg^−1^)	10,000.00	4100.00	7050.00
3	Biochar (kg^−1^)	10.20	0.125	5.46
4	Vermicompost (kg^−1^)	3.80	0.46	2.13
5	Lime (100 kg^−1^)	654.58	654.58	654.58
6	Seed (kg^−1^)	88.00	56.00	72.00
7	Labor (person⁻^1^ day^−1^)	200.00	250.00	225.00
8	Grain yield price (kg^−1^)	27.22	28.45	27.83
9	Straw yield price (kg^−1^)	4.30	4.56	4.43

Abbreviations: NB_a_ is the net benefit with the immediate lower total variable cost (TVC); NB_b_ is the net benefit with the next highest TVC; TVC_a_ is the immediate lower TVC; and TVC_b_ is the next highest TVC.

### Inputs and variable costs for partial analysis

A dry weight of 25.67 kg of old cow dung cost 20.59 Ethiopian Birr (ETB). The unit price of 1 t of vermicompost (VC) was 2,130.29 ETB t^−1^, calculated from total production costs (21,388.19 ETB ha^−1^) and mean VC applied over two years (10.04 t). Biomass conversion indicated that 1 local *quintal* (a traditional biomass unit) of old cow dung yields about 20.8 kg of VC (Table 2). Input prices were obtained from: (i) Burie district Agricultural Office for fertilizer and seed (average of three local retailers); (ii) actual production costs for biochar (maize cob collection, pyrolysis labor, transport); (iii) actual production costs for VC (earthworm procurement, feedstock, labor, maintenance); and (iv) local labor rates. Grain and straw prices were averaged from monthly market surveys during harvest (December–January) of 2023/24–2024/25. (Tables 3, 4,5, 6, 7, 8, 9, 10, 11, 12, 13, 14). The exchange-rate assumptions used in this study explicitly acknowledge exchange-rate volatility as a limitation and a sensitivity factor affecting comparisons across seasons. Economic analysis was based on the adjusted grain yields as described in Table 14.

**Table 3:** Baseline soil properties and characteristics of vermicompost and biochar.

Soil parameters	Value	Status	Critical level	Reference					
Soil physical Properties									
Sand (%)	50	–	–						
Silt (%)	14	–	–						
Clay (%)	36	–	–						
Textural class	Sandy clay	–	–						
Bulk Density (g cm^−3^)	1.41		< 1.40	^46^					
Soil porosity (%)	46.79	Low	> 50%	^47^					
Moisture content (%)	14	–							
Chemical Properties									
Exc. H (cmol₍c₎ kg^−1^)	0.88	–	–						
Exc. Al (cmol₍c₎ kg^−1^)	0.8	–	0.5						
Exc. Ac (cmol₍c₎ kg^−1^)	1.68	Moderate	2	^14^					
Soil pH (H_2_O) (1:2.5)	4.94	Very strongly acidic	6.6–7.3	^48^					
C-organic (%)	2.11	Low	4.0–10	^49^					
N-total (%)	0.25	Low	0.5	^39^					
C: N	8.44	–	–						
SOM (%)	3.63	Optimum	3.0–7.0	^48^					
avP (mg kg^−1^)	11.69	Very low	30–80	^48^					
Parameters	Vermicompost				Biochar				
	Year				Year				
	2023	2024	Mean	Rate	2023	2024	Mean	Rate	
pH	7.78	7.82	7.8	SlAl	10.14	9.63	9.88	StAl	^50^
OC (%)	8.29	13.56	10.9	High	65.46	63.78	64.62	High	^28,29^
TN (%)	2.16	2.68	2.42	VH	0.36	1.86	1.11	VH	^28,29^
avP (mg kg^−1^)	46.71	49.89	48.3	High	44.71	48.36	46.5	High	^49^
Color	Dark gray to black								

soil pH, soil reaction; Exc. Ac, exchangeable acidity; Exc. Al, exchangeable aluminum; Exc**.** H, exchangeable hydrogen; SOC, soil organic carbon; TN, total nitrogen; avP, available phosphorus; SC, sandy clay; pH, biochar and VC reactions; VH, very high; SlAl, slightly alkaline; StAl, strongly alkaline.

**Table 4:** Baseline soil chemical properties, mean values in T1, T14, and T24, and two-year changes.

	pH	Exc.Ac (cmol₍c₎ kg^−1^)	Exc.Al (cmol₍c₎ kg^−1^)	Exc.H (cmol₍c₎ kg^−1^)	TN (%)	avP (mg kg^−1^)	SOC (%)	SOM (%)
After T1	4.87	1.56	0.97	0.59	0.2	13.29	1.55	2.70
Baseline	4.94	0.88	0.8	0.88	0.25	11.69	2.11	3.69
Change	− 0.07	+ 0.68	+ 0.17	− 0.29	− 0.05	+ 1.6	− 0.56	− 0.99
After T14	5.54	0.96	0.52	0.43	0.30	39.14	2.64	4.60
Change	+ 0.6	+ 0.08	− 0.28	− 0.45	+ 0.05	+ 27.45	+ 24.29	+ 0.91
After T24	5.8	0.32	0.23	0.09	0.30	37.63	2.64	4.60
Change	+ 0.86	− 0.56	− 0.57	− 0.79	+ 0.05	+ 25.94	+ 0.53	+ 0.91

**Table 5:** Interaction effects of applied treatments on soil acidity parameters (mean ± SD).

Trt	Three-way interaction			Soil acidity parameters			
	N/P_2_O_5_ (kg ha^−1^)	BC (t ha^−1^)	VC (t ha^−1^)	pH_H_2_O (1:2.5)	Exc. Ac (cmol₍c₎ kg^−1^)	Exc. Al (cmol₍c₎ kg^−1^)	Exc. H (cmol₍c₎ kg^−1^)
T1	0	0	0	4.87^pq^ ± 0.01	1.56^b^ ± 0.032	0.97^b^ ± 0. 035	0.59^c^ ± 0.015
T2	0	0	5.02	5.16^ m-o^ ± 0.01	1.25^ cd^ ± 0.001	0.69^ fg^ ± 0.010	0.56^ cd^ ± 0.012
T3	0	0	10.04	5.21^ l-n^ ± 0.020	1.15^e^ ± 0.02	0.62^hi^ ± 0.015	0.52^de^ ± 0.005
T4	120/69	0	0	5.01^op^ ± 0.02	1.54^b^ ± 0.05	0.89^c^ ± 0.03	0.65^b^ ± 0.02
T5	120/69	0	5.02	5.25^ k-n^ ± 0.017	1.30^c^ ± 0.012	0.82^d^ ± 0.001	0.48^ef^ ± 0.001
T6	120/69	0	10.04	5.30^j-m^ ± 0.02	1.21^d^ ± 0.005	0.75^e^ ± 0.12	0.45^ fg^ ± 0.005
T7	240/138	0	0	4.75^q^ ± 0.012	1.92^a^ ± 0.072	1.15^a^ ± 0.08	0.76^a^ ± 0.057
8	240/138	0	5.02	5.37^ik^ ± 0.025	1.11^e^ ± 0.021	0.81^d^ ± 0.049	0.30^ k-m^ ± 0.069
T9	240/138	0	10.04	5.44^ g-j^ ± 0.011	0.94^gh^ ± 0.021	0.72^ef^ ± 0.01	0.22^o^ ± 0.025
T10	0	4	0	5.30^j-m^ ± 0.02	0.99^ fg^ ± 0.012	0.65^ fg^ ± 0.012	0.34^jk^ ± 0.0001
T11	0	4	5.02	5.38^ h-k^ ± 0.012	0.92^hi^ ± 0.010	0.60^ij^ ± 0.005	0.31^kl^ ± 0.011
T12	0	4	10.04	5.45^ g-j^ ± 0.010	0.86^ij^ ± 0.015	0.57^jk^ ± 0.005	0.28^ l-n^ ± 0.011
T13	120/69	4	0	5.53^d-I^ ± 0.47	1.04f. ± 0.025	0.55^kl^ ± 0.005	0.49^ef^ ± 0.02
T14	120/69	4	5.02	5.54^d-h^ ± 0.03	0.96^gh^ ± 0.020	0.52^ lm^ ± 0.012	0.43^gh^ ± 0.01
T15	120/69	4	10.04	5.51^e-I^ ± 0.026	0.87^ij^ ± 0.012	0.49^ mn^ ± 0.012	0.37^ij^ ± 0.01
T16	240/138	4	0	5.12^no^ ± 0.015	0.88^ij^ ± 0.005	0.47^no^ ± 0.12	0.40^hi^ ± 0 0.015
T17	240/138	4	5.02	5.21^ l-n^ ± 0.02	0.84^jk^ ± 0.025	0.45^op^ ± 0.005	0.39^hi ±^ 0.02
T18	240/138	4	10.04	5.64^b-e^ ± 0.09	0.79^ k^ ± 0.152	0.42^pq^ ± 0.005	0.37^ij^ ± 0.017
T19	0	8	0	5.32^j-l^ ± 0.03	0.68^ l^ ± 0.015	0.39^qr^ ± 0.005	0.28^ l-n^ ± 0.011
T20	0	8	5.02	5.38^i-k^ ± 0.03	0.63^ l^ ± 0.017	0.37^rs^ ± 0.0001	0.26^ m-o^ ± 0.02
T21	0	8	10.04	5.82^a^ ± 0.010	0.56^ m^ ± 0.010	0.33^st^ ± 0.012	0.22^o^ ± 0.005
T22	120/69	8	0	5.68^a-d^ ± 0.07	0.54^ m^ ± 0.025	0.30^t^ ± 0.012	0.24^no^ ± 0.017
T23	120/69	8	5.02	5.74^a-c^ ± 0.06	0.42^n^ ± 0.055	0.26^u^ ± 0.026	0.16^p^ ± 0.030
T24	120/69	8	10.04	5.80^ab^ ± 0.006	0.32^o^ ± 0.028	0.23^u^ ± 0.001	0.09^qr ±^ 0.028
T25	240/138	8	0	5.48f.^-I^ ± 0.021	0.33^o^ ± 0.03	0.17^v^ ± 0.001	0.16^p^ ± 0.031
T26	240/138	8	5.02	5.55^d-g^ ± 0.06	0.21^p^ ± 0.091	0.10^w^ ± 0.034	0.11^q^ ± 0.056
T27	240/138	8	10.04	5.62^c-f^ ± 0.05	0.12^q^ ± 0.089	0.05^x^ ± 0.045	0.06^r^ ± 0.061
Mean				5.38	0.889	0.534	0.35
F-Test				< 0.0001	< 0.0001	< 0.0001	< 0.0001
LSD (0.05)				0.053***	0.02***	0.0134***	0.0156***
R^2^				0.92	0.99	0.99	0.98
CV (%)				1.79	4.11	4..59	8.07

Trt, treatments; LSD, least significant difference; BC, maize cob biochar; CV, coefficient of variation (%); *** Significant at the 0.001 probability level; DF, degree of freedom. The means in the column within a parameter followed by the same letter(s) are not significantly different at *P* = 0.05.

**Table 6:** Interaction effects of applied treatments on soil fertility parameters (mean ± SD).

Trt	Three-way interaction			Soil fertility parameters			
	N/P_2_O_5_ (kg ha^−1^)	BC (t ha^−1^)	VC (t ha^−1^)	Total N (%)	avP (mg kg⁻^1^)	SOC (%)	SOM (%)
T1	0	0	0	0.20^ l^ ± 0.0057	13.29^r^ ± 2.55	1.55^ m^ ± 0.01	2.70^n^ ± 0.017
T2	0	0	5.02	0.26^i-k^ ± 0.0057	26.66^ m-o^ ± 1.11	2.43^j^ ± 0.02	4.23^kl^ ± 0.034
T3	0	0	10.04	0.29^c-f^ ± 0.0001	28.81^ lm^ ± 0.88	2.58^d^ ± 0.001	4.49^de^ ± 0.001
T4	120/69	0	0	0.28f.^-h^ ± 0.015	30.30^kl^ ± 0.81	2.516^ h^ ± 0.005	4.38^hi^ ± 0.01
T5	120/69	0	5.02	0.28^e-h^ ± 0.023	30.45^j-l^ ± 0.68	2.56^d-f^ ± 0.001	4.46 ^e–g^ ± 0.001
T6	120/69	0	10.04	0.30^b-d^ ± 0.011	31.72^jk^ ± 0.27	2.586^de^ ± 0.005	4.49^ef^ ± 0.01
T7	240/138	0	0	0.31^a-c^ ± 0.015	32.98^i-k^ ± 0.42	2.64^ab^ ± 0.005	4.59^ab^ ± 0.01
8	240/138	0	5.02	0.32^ab^ ± 0.02	36.03f.^-h^ ± 1.84	2.64^ab^ ± 0.005	4.60^ab^ ± 0.01
T9	240/138	0	10.04	0.32^ab^ ± 0.0115	38.46^d-f^ ± 1.74	2.65^ab^ ± 0.02	4.61^ab^ ± 0.036
T10	0	4	0	0.24^ k^ ± 0.0001	21.48^q^ ± 1.33	2.36^ k^ ± 0.001	4.11^ m^ ± 0.001
T11	0	4	5.02	0.25^jk^ ± 0.0057	23.14^pq^ ± 0.67	2.45^kl^ ± 0.006	4.27^jk^ ± 0.01
T12	0	4	10.04	0.27^ g-j^ ± 0.0001	24.74^op^ ± 0.76	2.51^ h^ ± 0.006	4.38^i^ ± 0.01
T13	120/69	4	0	0.27f.^-i^ ± 0.0057	27.90^ l-n^ ± 0.74	2.46^i^ ± 0.01	4.29^j^ ± 0.017
T14	120/69	4	5.02	0.30^b-e^ ± 0.017	39.14^de^ ± 2.57	2.64^ab^ ± 0.005	4.60^ab^ ± 0.01
T15	120/69	4	10.04	0.322^a^ ± 0.0.0115	41.01^ cd^ ± 2.70	2.653^a^ ± 0.005	4.62^a^ ± 0.01
T16	240/138	4	0	0.30^b-d^ ± 0.0115	34.75^hi^ ± 0.86	2.63^bc^ ± 0.015	4.58^bc^ ± 0.026
T17	240/138	4	5.02	0.31^ab^ ± 0.02	41.93^c^ ± 1.15	2.64^ab^ ± 0.015	4.59^ab^ ± 0.026
T18	240/138	4	10.04	0.32^ab^ ± 0.0057	39.97^c-e^ ± 4.67	2.65^ab^ ± 0.006	4.61^ab^ ± 0.011
T19	0	8	0	0.27^ h-j^ ± 0.0057	25.56^n-p^ ± 0.89	2.413^j^ ± 0.05	4.20^ l^ ± 0.09
T20	0	8	5.02	0.28f.^-h^ ± 0.0057	26.36^ m-o^ ± 0.49	2.52^hi^ ± 0.01	4.39^hi^ ± 0.02
T21	0	8	10.04	0.28f.^-h^ ± 0.0001	28.97^ lm^ ± 0.56	2.54^ fg^ ± 0.008	4.43^gh^ ± 0.014
T22	120/69	8	0	0.28f.^-h^ ± 0.0001	30.27^kl^ ± 0.88	2.55^ef^ ± 0.011	4.45^ fg^ ± 0.02
T23	120/69	8	5.02	0.28^e-h^ ± 0.0057	33.17^ij^ ± 1.15	2.57^e-g^ ± 0.01	4.48^e-g^ ± 0.017
T24	120/69	8	10.04	0.30^b-d^ ± 0.0115	37.63^e-g^ ± 1.63	2.64^ab^ ± 0.005	4.60^ab^ ± 0.01
T25	240/138	8	0	0.29^d-g^ ± 0.0057	35.16^ g-I^ ± 1.62	2.58^ef^ ± 0.006	4.49^ef^ ± 0.012
T26	240/138	8	5.02	0.31^ab^ ± 0.02	45.34^b^ ± 2.63	2.64^ab^ ± 0.005	4.61^ab^ ± 0.01
T27	240/138	8	10.04	0.30^b-d^ ± 0.023	48.38^a^ ± 1.61	2.60^ cd^ ± 0.032	4.54^ cd^ ± 0.056
Mean				0.28	32.36	2.53	4.40
F-Test				0.001	< 0.00001	< 0.0001	< 0.0001
LSD (0.05)				0.0064**	0.9153***	0.0084***	0.0147***
R^2^				0.88	0.97	0.996	0.996
CV (%)				4.12	5.18	0.61	0.61

LSD, least significant difference; BC, biochar, CV, coefficient of variation (%); ***, **, Significant at the 0.001 and 0.01 probability level; DF, degree of freedom. The means in the column within a parameter followed by the same letter(s) are not significantly different at *P* = 0.05.

**Table 7:** Mean squares and significance of integrated applications of inorganic N/P₂O₅, vermicompost (VC), and biochar (BC) on soil acidity and fertility parameters after two years (2023–2025).

Source of variation	DF	pH	Exc. H	Exc. Ac	avP	SOC	SOM	TN
Block	2	0.022 ns	0.0007 ns	0.0001 ns	2.73 ns	0.0002 ns	0.0005 ns	0.00038 ns
N/P_2_O_5_	2	0.203***	0.0385***	0.1886***	1523.19***	0.4883***	1.4819***	0.01582***
BC	2	1.368***	0.7367***	5.5652***	150.00***	0.0890***	0.2700***	0.00021 ns
VC	2	0.405**	0.1514***	0.6230***	415.67***	0.2785***	0.8453***	0.00609***
N/P_2_O_5_ × BC	4	0.035**	0.0412***	0.1060***	36.57***	0.0803***	0.2436***	0.00100***
N/P_2_O_5_ × VC	4	0.067***	0.0185***	0.0343***	2.61 ns	0.1232***	0.3738***	0.00060**
BC × VC	4	0.123***	0.0336***	0.1569***	4.94 ns	0.0631***	0.1914***	0.00032 ns
N/P_2_O_5_ × BC × VC	8	0.081***	0.0193***	0.0396***	50.12***	0.0899***	0.2728***	0.00055**
Error	52	0.009	0.0008	0.0013	2.81	0.0002	0.0007	0.00014
	80	80	80	80	80	80	80	80
R-Square		0.92	0.982	0.995	0.970	0.996	0.996	0.887
Coeff Var		1.79	8.078	4.11	5.18	0.612	0.612	4.124
Root MSE		0.096	0.0286	0.0365	1.1676	0.015	0.0269	0.012
Mean		5.38	0.355	0.889	32.36	2.526	4.400	0.286

DF, degrees of freedom; pH, soil reaction; Exc. H, exchangeable hydrogen. Exc. Ac, exchangeable acidity; avP, available phosphorus; SOC, soil organic carbon; SOM, soil organic matter, TN, soil total nitrogen; ***, **, *, Significant at 0.001, 0.01, and 0.05 probability level, respectively and ns, non-significant at P = 0.05.

**Table 8:** Pearson correlation coefficients (*r*) between selected soil chemical properties and maize grain yield.

	pH	TN (%)	avP (mg kg^-1^)	SOC (%)	SOM (%)	Exc. H (cmol₍c₎ kg^−1^)	Exc. Al (cmol₍c₎ kg^−1^)	Exc. Ac (cmol₍c₎ kg^−1^)	GY (t ha^-1^)
pH	1	0.271 ns	0.354 ns	0.395*	0.404*	− 0.780**	0.091 ns	− 0.945**	0.529**
TN (%)	0.271 ns	1	0.874**	0.866**	0.865**	− 0.316 ns	0.17 ns	− 0.267 ns	0.688**
avP (mg kg^-1^)	0.354 ns	0.4 4**	1	0.729**	0.731**	− 0.369 ns	0.194 ns	− 0.316 ns	0.649**
SOC (%)	0.395*	0.866**	0.729**	1	1.000**	− 0.450*	0.202 ns	− 0.416*	0.693**
SOM (%)	0.404*	0.865**	0.731**	1.000**	1	− 0.458*	0.205 ns	− 0.424*	0.694**
Exc. H (cmol₍c₎ kg^−1^)	− 0.945**	− 0.316 ns	− 0.369 ns	− 0.450*	− 0.458*	1	− 0.649**	0.748**	− 0.577**
Exc. Al (cmol₍c₎ kg^−1^)	0.091 ns	0.17 ns	0.194 ns	0.202 ns	0.205 ns	− 0.649**	1	0.019 ns	0.284 ns
Exc. Ac (cmol₍c₎ kg^−1^)	− 0.945**	− 0.267 ns	− 0.316 ns	− 0.416*	− 0.424*	0.748**	0.019 ns	1	− 0.511**
GY (t ha^-1^)	0.529**	0.688**	0.649**	0.693**	0.694**	− 0.577**	0.284 ns	− 0.511**	1

pH, soil reaction; TN, total nitrogen; avP, available phosphorus; Exc. Ac, exchangeable acidity; Exc. Al, exchangeable aluminum; Exc. H, exchangeable hydrogen; SOC, soil organic carbon; SOM, soil organic matter; GY, grain yield. *, **, ***, correlation significant at *P* ≤ 0.05, 0.01, and 0.001, respectively; ns, nonsignificant.

**Table 9:** Kaiser–Meyer–Olkin (KMO) measure of sampling adequacy and Bartlett’s sphericity of test.

KMO and Bartlett’s Test		
Kaiser‒Meyer‒Olkin measure of sampling adequacy		0.663
Bartlett’s test of sphericity	Approx. Chi-Square	475.51
	df	36
	Sig	< 0.001

**Table 10:** ANOVA for maize grain yield using PROC MIXED.

Source of variations	Num DF	Den DF	F Value	P-value	Estimate/Notes
Fixed effects					
N/P_2_O_5_ rate (NP)	2	8	12.35	0.003	Significant effect on grain yield
BC	2	8	15.42	0.001	Significant effect on grain yield
VC	2	8	18.227	< 0.001	Significant effect on grain yield
NP × BC	4	8	6.12	0.012	Significant 2-way-interaction
NP × VC	4	8	3.45	0.062	Not significant
BC × VC	4	8	2.78	0.097	Not significant
NP × BC × VC	8	8	4.15	0.027	Significant 3-wayinteraction
Random effects					Variance (t^2^ / ha^2^)
Year	–	–	–	–	0.8123 (variation between years)
Replication (Year)	–	–	–	–	0.4578 (variations among replications within years)
Residual	–	–	–	–	1.2356 (Unexplained experimental error)

BC, biochar; VC, vermicompost; Num DF, numerator degrees of freedom; Den DF, denominator degrees of freedom; t, tonne; ha, hectare.

**Table 11:** Performance metrics of yield prediction models.

Metrics	N	Minimum	Maximum	Mean	Mean std. error	Std. deviation
MAE (t ha^−1^)	81	0.00	2.97	0.92	0.072	0.65
RMSE (t ha^−1^)	81	0.00	2.97	0.92	0.072	0.65
MSE (t ha^−1^)	81	− 2.10	2.97	0.00	0.125	1.13
MPE %	81	− 24.42	23.34	− 1.28	1.29	11.61
MAPE %	81	0.01	24.42	9.78	0.69	6.28
CV	81			9.92		
NSE	81			0.62		

N, number of observations; MAE, Mean Absolute Error; RMSE, Root Mean Square Error; MSE, Mean Square Error; MPE, Mean Percentage Error, MAPE, Mean Absolute Percentage Error; NSE, Nash–Sutcliffe Efficiency; NSE, Nash–Sutcliffe Efficiency; CV, coefficient of variation.

**Table 12:** ANOVA Table, coefficients of multiple linear regression and interpretation of the model.

ANOVA^a^								
	Model	Sum of Squares	df	Mean Square	F	Sig		
1	Regression	163.459	9	18.162	13.506	0.000^b^		
	Residual	86.064	64	1.345				
	Total	249.523	73					
Coefficients								
Model	Unstandardized Coefficients			Standardized Coefficients	t-statistics	Sig	Collinearity Statistics	Standardized Coefficients
		B	Std. Error	Beta			Tolerance	VIF
1	(Constant t ha^−1^)	− 20.792	5.136		− 4.048	0.000		
	N/P_2_O_5_ rate	0.535	0.419	0.237	1.276	0.207	0.156	6.406
	BC rate	0.879	0.478	**0.389**	1.837	0.071	0.120	8.312
	VC rate	0.092	0.317	0.041	0.289	0.773	0.274	3.647
	Soil pH	2.848	0.891	**0.428**	3.197	0.002	0.301	3.321
	Soil TN	12.145	11.608	0.186	1.046	0.299	0.170	5.880
	Soil avP	0.017	0.051	0.072	0.333	0.740	0.114	8.758
	Soil OC	2.301	1.179	0.271	1.951	0.055	0.280	3.572
Statistics model summary ^b^	Value							
R = 0.809	Strong to very strong correlation between observed and predicted values							
R Square = 0.655	Our model explains 65.5% of the variation in grain yield							
Adjusted R Square = 0.607	Adjusted for number of predictors, this is still fairly very strong in field research with complex variability							
Durbin-Watson = 1.032	Indicated no-auto-correlation between independent variables, accepted and satisfied linear regression assumption. It is in the acceptable range value (< 2.5)							

df, degrees of freedom; VIF, Variance Inflation (VIF < 10); a, dependent variable (grain yield); b, predictors: (Constant), Exc. H, NP, VC, SOC, pH, BC, TN, avP.

Significant values are in bold.

**Table 13:** Sensitivity analysis of maize grain yield over two growing seasons.

Scenario	Grain price change (%)	Fertilizer price change (%)	Labor cost change (%)	Net benefit (ETB ha^−1^)	MRR (%)	Recommendation
Baseline	0	0	0	289,124	149.1%	Profitable
Scenario 1	− 20	0	0	227,891	117.4	Profitable
Scenario 2	+ 20	0	0	350,357	180.8	Profitable
Scenario 3	0	+ 20	0	279,246	125.3	Profitable
Scenario 4	0	0	+ 20	284,877	138.2	Profitable
Scenario 5	− 10	+ 10	+ 10	256,432	102.7	Profitable

**Table 14:** Partial budget analysis of maize yield for determining economically profitable combinations of N/P₂O₅, biochar (BC), and vermicompost (VC) over two growing seasons (2023/24 and 2024/25).

Treatments	N/P_2_O_5_ (kg ha^−1^)	Biochar (t ha^−1^)	VC (t ha^−1^)	AGY GY (kg ha^−1^)	ASY (kg ha^−1^)	GB (ETB ha^−1^)	Mean TVC(ETB ha^−1^)	NB(ETB ha^−1^)	MRR (%)
T1	0	0	0	4170.68	8211.00	156,473.24	22,694.20	128,546.20	–
T10	0	4	0	7570.42	11,660.83	262,077.38	44,094.20	216,834.10	413
T2	0	0	0.50	6279.72	9074.97	215,815.68	46,225.05	167,288.50	− 2325.16D
T4	120/69	0	0	7219.90	9447.00	241,732.76	48,549.72	192,486.65	− 546.46D
T19	0	8	0	7242.75	14,221.07	269,492.52	65,444.20	197,914.70	− 88.66 D
T11	0	4	0.50	7870.17	11,884.67	269,138.39	67,625.05	202,630.25	− 60.36D
T3	0	0	10.04	6835.23	10,034.25	239,638.82	69,755.90	163,503.90	− 207.8D
T13	120/69	4	0	9893.83	11,437.23	326,018.82	69,949.72	254,047.30	143.93
T5	120/69	0	5.04	8192.47	9656.63	270,944.29	72,080.57	197,037.80	− 2675.43D
T7	240/138	0	0	8125.35	9725.52	265,101.37	73,205.16	193,957.95	− 1845.81D
T20	0	8	5.04	7708.90	11,540.57	268,727.13	88,975.05	175,104.95	− 413.93D
T12	0	4	10.04	9605.55	11,430.43	294,839.88	91,155.90	224,789.70	− 137.97D
T22	120/69	8	0	8726.05	12,025.77	293,950.73	91,299.72	202,984.45	− 239.17 D
T14	120/69	4	5.02	11,566.85	14,102.17	376,565.94	93,480.57	289,124.10	149.067
T16	240/138	4	0	10,978.55	11,437.55	349,927.50	94,605.16	259,307.25	− 2651.35D
T6	120/69	0	10.04	8105.05	10,277.20	269,576.53	95,611.42	173,719.90	− 11,468.6D
T8	240/138	0	5.02	9424.77	10,038.48	302,354.54	96,736.01	208,110.55	− 2488.36D
T17	240/138	4	5.02	11,169.63	11,912.87	353,117.83	110,153.01	251,050.60	− 228.36D
T21	0	8	10.04	9044.20	10,830.47	295,056.13	112,505.90	185,156.65	− 546.47D
T23	120/69	8	5.02	10,290.42	11,969.65	339,147.26	114,830.57	222,452.15	− 312.28D
T25	240/138	8	0	8927.22	14,624.85	312,246.75	115,955.16	195,766.00	− 415.39D
T15	120/69	4	10.04	10,654.92	14,007.10	354,327.23	117,011.42	239,743.25	− 209.86D
T9	240/138	0	10.04	9771.08	10,063.78	314,128.63	120,266.86	194,325.90	− 353.91D
T24	120/69	8	10.04	11,536.00	14,064.73	368,168.20	138,361.42	242,439.75	− 104.02D
T26	240/138	8	5.02	8656.50	14,669.63	305,812.28	139,486.01	164,878.15	− 270.07D
T18	240/138	4	10.04	10,747.9	11,706.55	348,612.7	141,666.85	207,248.25	− 169.92 D
T27	240/138	8	10.04	8182.93	14,648.36	294,412.6	163,016.85	128,118.1	− 231.54 D

AGY, adjusted grain yield; ASY, adjusted straw yield; GB, gross benefit; TVC, total variable cost; D, Dominated treatments and MRR, marginal rate of return and NB, net benefit. Maize yield was calculated on the selling price of maize at 27.22 ETB kg^−1^) (2024) and 28.45 ETB (2025) cropping season (mean 27.83 ETB kg^−1^ of maize).

### Regression model

The effects of inorganic N/P_2_O_5_nutrient, BC, and VC on soil properties and maize grain yield were analyzed using a linear mixed-effects model via the PROC MIXED procedure in SAS version 9.4^45^. The model was specified as:

7 $$\begin{aligned} {\mathrm{Y}}_{{{\mathrm{ijklm}}}} = & \mu + {\mathrm{NP}}_{{\mathrm{i}}} + {\mathrm{BC}}_{{{\mathrm{j}}}} + {\mathrm{VC}}_{{{\mathrm{k}}}} + {\mathrm{Y}}_{{{\mathrm{l}}}} + ({\mathrm{NPBC}})_{{{\mathrm{ij}}}} + ({\mathrm{NPVC}})_{{{\mathrm{ik}}}} \\ \quad & + ({\mathrm{BCVC}})_{{{\mathrm{jk}}}} + ({\mathrm{NPBCVC}})_{{{\mathrm{ijk}}}} + \left( {{\mathrm{NPY}}} \right)_{{{\mathrm{il}}}} + \left( {{\mathrm{BCY}}} \right)_{{{\mathrm{jl}}}} \\ \quad & + \left( {{\mathrm{VCY}}} \right)_{{{\mathrm{kl}}}} + {\text{ R}}_{{\mathrm{m}}} ({\mathrm{Yl}}) + \varepsilon_{{{\mathrm{ijklm}}}} \\ \end{aligned}$$

Abbreviations: Y_ijklm_, response variable (soil property or grain yield); μ, overall mean; NP_i_ , fixed effect of inorganic N/P_2_O_5_ nutrient rate (i = 0/0, 120/69, 240/138 kg ha^−1^); BC_j_, fixed effect of biochar rate (j = 0, 4, 8 t ha^−1^); VC_k_, fixed effect of vermicompost rate (k = 0, 5.02, 10.04 t ha^−1^);Y_l_ , random effect of year (l = 2023/24, 2024/25);NPBC_*ij*_*,* NPVC_*ik*_*,* BCVC_jk_, two-way interaction terms among fixed factors; NBV_ijk_, three-way interaction term among fixed factors; NPY_*il*_, BCY_jl_, VCY_kl,_ interactions between fixed factors and year; R_m_(Y_l_), random effect of replication (m = 1, 2, 3) nested within year; ε_ijklm_​, experimental error.

### Factor analysis

Factor analysis was employed to reduce the dimensionality of the dataset by summarizing correlated variables into a smaller number of latent factors while retaining the maximum shared variance. The extracted factor scores were subsequently used for further analysis as composite indices representing the original variables. Sampling adequacy was assessed using the Kaiser–Meyer–Olkin (KMO) statistic, which evaluates the proportion of variance attributable to common factors and the extent of partial correlations among variables. KMO values were interpreted as follows: < 0.50 (poor), 0.50–0.59 (marginal), 0.60–0.69 (acceptable), and ≥ 0.70 (good). Bartlett’s test of sphericity was applied to examine whether the correlation matrix significantly differed from an identity matrix, indicating sufficient intercorrelation among variables for factor extraction. A significant result (P < 0.05) was considered evidence supporting the suitability of the data for factor analysis. For factor analysis degrees of freedom was calculated using Eq. 8

8 $$df = \frac{{p\left( {p - 1} \right)}}{2}$$

Abbreviations: df, degrees of freedom; p, number of variables included in the correlation matrix (n = 9).

### Statistical data analysis

All data were subjected to analysis of variance (ANOVA) using the PROC MIXED procedure in SAS version 9.4^45^. In the mixed model, inorganic N/P_2_O_5_ nutrient rates (0, 120/69, and 240/138 kg ha^−1^), biochar rates (0, 4, and 8 t ha^−1^), and vermicompost rates (0, 5.02, and 10.04 t ha^−1^) were considered fixed factors, while year (2023/24 and 2024/25) and replication nested within year were treated as random effects to account for environmental variation across growing seasons. Normality and homogeneity of variance were verified using the Shapiro–Wilk test^51^ and Levene’s test^52^, respectively. Following a significant F-test (P ≤ 0.05), treatment means were separated using Tukey’s Honestly Significant Difference (HSD) test^53,54^. This post hoc procedure was selected because it controls the family-wise error rate at the 5% significance level across all pairwise comparisons, which is particularly important given the 27 treatment combinations in this factorial experiment. When the year × N/P₂O₅ × BC × VC interaction was significant, results were presented separately by year; however, when the year × treatment interaction was not significant, results were presented as combined treatment means across years. Pearson correlation coefficients (r) were calculated via SAS version 9.4 to evaluate the relationships between grain yield and the selected soil chemical properties. Correlation strengths were interpreted via standard guidelines: no correlation (0 < r < 0.2), weak correlation (0.2 ≤ r < 0.4), moderate correlation (0.4 ≤ r < 0.6), strong correlation (0.6 ≤ r < 0.8), and very strong correlation (r ≥ 0.8). The interaction plot was generated using OriginPro 2024.

## Results

### Background soil characteristics and treatment effects on soil chemical properties

The initial soil analysis revealed acidity and fertility limitations (Table 3). Results of paired-sample t-tests for pre- and post-soil properties are shown in Supplementary Table S4. The initial soil characterization was based on a single composite sample from 10 random locations across the experimental field, following standard practice for establishing baseline conditions^37^. While this approach provides an estimate of mean initial conditions, it does not capture fine-scale spatial variability that may have existed prior to treatment application. The randomized complete block design with three replications helps account for spatial variation by blocking, but pre-treatment sampling of individual plots would have provided more precise baseline data for calculating treatment-induced changes.

The soil was classified as sandy clay, with a mean bulk density (BD) of 1.41 g cm^−3^, suggesting that both physical and chemical constraints may restrict productivity and therefore require appropriate soil amendment interventions. The initial soil also exhibited characteristics typical of very strongly acidic Nitisols in the Ethiopian highlands^48^ ). Low pH (4.94), elevated exchangeable Al^3^⁺ (0.8 cmol₍c₎ kg^−1^), low available P (11.69 mg kg^−1^, Mehlich-3), and moderate exchangeable acidity (1.68 cmol₍c₎ kg^−1^) (Table 4). Considering that toxicity risk in maize typically begins at exchangeable Al^3^⁺ levels of ≥ 0.5 cmol₍c₎ kg^−1^ (marginal toxicity), with moderate toxicity occurring at 0.5–2.0 cmol₍c₎ kg^−1^ and high toxicity above 2.0 cmol₍c₎ kg^−1^, the measured Al^3^⁺ at the experimental site indicates a potential risk of aluminum toxicity^55^. This confirms that soil acidity and nutrient limitations were significant constraints for maize growth^55^.

Soil organic carbon content was low (2.11%), the TN content was 0.25%, and the avP contents) were very low at 11.69 mg kg^−1^. Although soi OM content was within the optimum range (3.63%), the overall nutrient status was inadequate for optimal maize production. At the end of the pyrolysis process, 89.1 kg of BC was obtained from 212.34 kg of maize cobs in 2023/24, corresponding to a yield of 41.96%, and 89.1 kg from 208.49 kg of maize cobs in 2024/25, corresponding to a yield of 42.73%. The average biochar yield over the two seasons was 42.35%. The BC had a pH of 9.88, an OC of 64.62%, a TN of 1.11%, and an avP of 46.5 mg kg^−1^, indicating that high-quality BC is suitable as a liming material (Table 4). For BC, especially when it is produced from woody residues, crop residues, or high-temperature pyrolysis, the C:N ratios commonly range from 30:1 to > 100:1^56^. The high C:N ratio (58:1) reflects the recalcitrant nature of the biochar and suggests limited short-term nitrogen availability, which is typical of BC produced from lignocellulosic feedstocks. The VC applied at planting was mature and well decomposed, with a mean pH of 7.80, an OC of 10.9%, a TN of 2.42%, a narrow C:N ratio of 4.5:1, and an avP of 48.3 mg kg^−1^. The low C:N ratio suggests rapid nitrogen release, and the physical characteristics of the VC were favorable (nongranular, no foul odor), highlighting its potential to improve soil fertility (Table 4).

### ***Effects of N/P***_***2***_***O***_***5***_***, BC, and VC rates and their combinations on soil acidity and fertility***

Soil pH and Exc. Ac was highly significantly affected (P < 0.0001) by the three-way effects of N/P₂O₅, BC, VC (Tables 5 and 7). Following the uniform application of lime across all treatments, soil pH ranged from 4.75 in plots receiving only 240/138 kg N/P₂O₅ ha^−1^ to 5.82 in plots treated with the combined application of 8 t BC ha^−1^ + 10.04 t VC ha^−1^. Exchangeable Al and H were also significantly reduced by BC and VC, applied alone or in combination with N/P₂O₅, relative to the limed control baseline, although the magnitude of reduction varied with amendment type and rate (Tables 5 and 7). Exchangeable Al decreased by 40–66%, and Exc. Ac decreased by 19–65%, with the greatest reduction observed in plots receiving 8 t BC ha^−1^ + 10.04 t VC ha^−1^ + 120/69 kg N/P₂O₅ ha^−1^, compared with 240/138 kg N/P₂O₅ ha^−1^ alone under the same limed condition. These results showed the additional effectiveness of integrated organic and inorganic amendments in further ameliorating soil acidity beyond the baseline effect of lime.

**Fig. 3:**
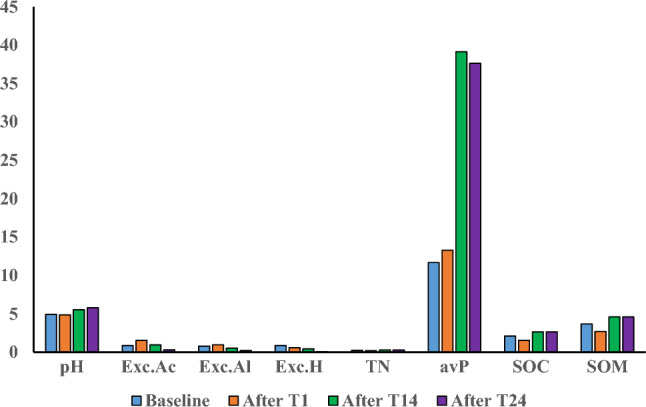
Changes in soil chemical properties after two years under the combined effects of N/P₂O₅, BC, and VC compared to baseline conditions.

Soil TN was significantly influenced (P = 0.001) by N/P₂O₅, BC, VC, and their combinations under the uniform limed soil condition, ranging from 0.20% in the limed control to 0.322% in the plots receiving 4 t BC ha^−1^ + 5.02 t VC ha^−1^ + 120/69 kg N/P₂O₅ ha^−1^, representing a 37.88% increase relative to the limed baseline (Tables 6 and 7). The avP was highly significantly affected (P < 0.0001) by the main effects and three-way interactions of N/P₂O₅, BC, and VC under the same limed condition, ranging from 13.29 mg kg^−1^ in the limed control to 48.38 mg kg^−1^ in plots receiving 8 t BC ha^−1^ + 10.04 t VC ha^−1^ + 120/69 kg N/P₂O₅ ha^−1^, a 72.53% increase over the limed baseline. Both individual and combined applications of N/P₂O₅, BC, and VC consistently enhanced soil avP beyond the effect of lime alone. Soil organic carbon and soil SOM were highly significantly affected (*P* < 0.0001) by N/P₂O₅, BC, VC, and their interactions under the limed condition (Table 6 and Table 7). The SOC content ranged from 1.55% to 2.65%, and the SOM content ranged from 2.70% to 4.62%, with the highest values observed in plots receiving 4 t BC ha^−1^ + 5.02 t VC ha^−1^ + 120/69 kg N/P₂O₅ ha^−1^. The application of BC and VC, individually or in combination with inorganic fertilizers, improved SOC and overall soil quality relative to the limed baseline, likely due to improved soil acidity conditions. Changes in soil chemical properties for the control, T14, and T24, relative to baseline conditions (Table 4 and Fig. 3). Any unaccounted spatial soil quality variability would increase experimental error and make treatment differences harder to detect using SAS (2023). The fact that we observed highly significant treatment effects (P < 0.01) with low CVs suggests that spatial variability was adequately controlled through blocking and replication.

Paired-samples test comparing soil chemical properties before the experiment and after two years are illustrated in Table 4 (selected treatments) and Supplementary Table S4. A larger absolute t-statistic value indicates a stronger difference between the paired observations.

### Effects of N/P₂O₅ nutrient, BC, and VC rates and their combinations on grain yield

**Fig. 4:**
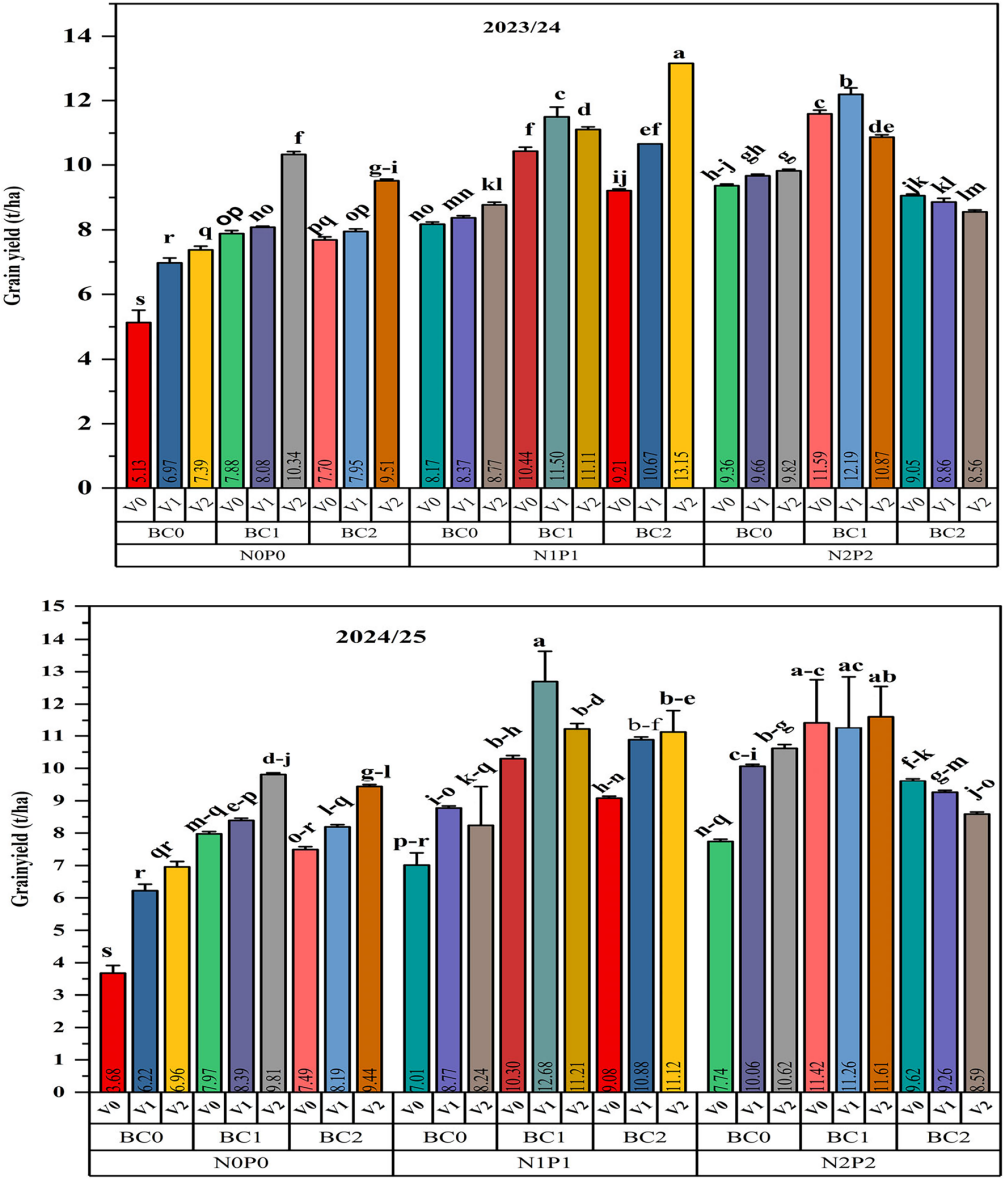
Three-way interaction effects of N/P_2_O_5_ inorganic nutrients, biochar (BC), and vermicompost (VC) on maize grain yield in 2023/24 and 2024/25. Bars represent mean values ± standard error, and different letters indicate significant differences at *P* < 0.05 (Tukey’s HSD). Where: N0P0, N1P1 and N2P2 were three rates of N/P_2_O_5_ nutrients, BC0, BC1, BC2; V0; V1 and V2 were three rates of BC and VC applied, respectively.

**Fig. 5:**
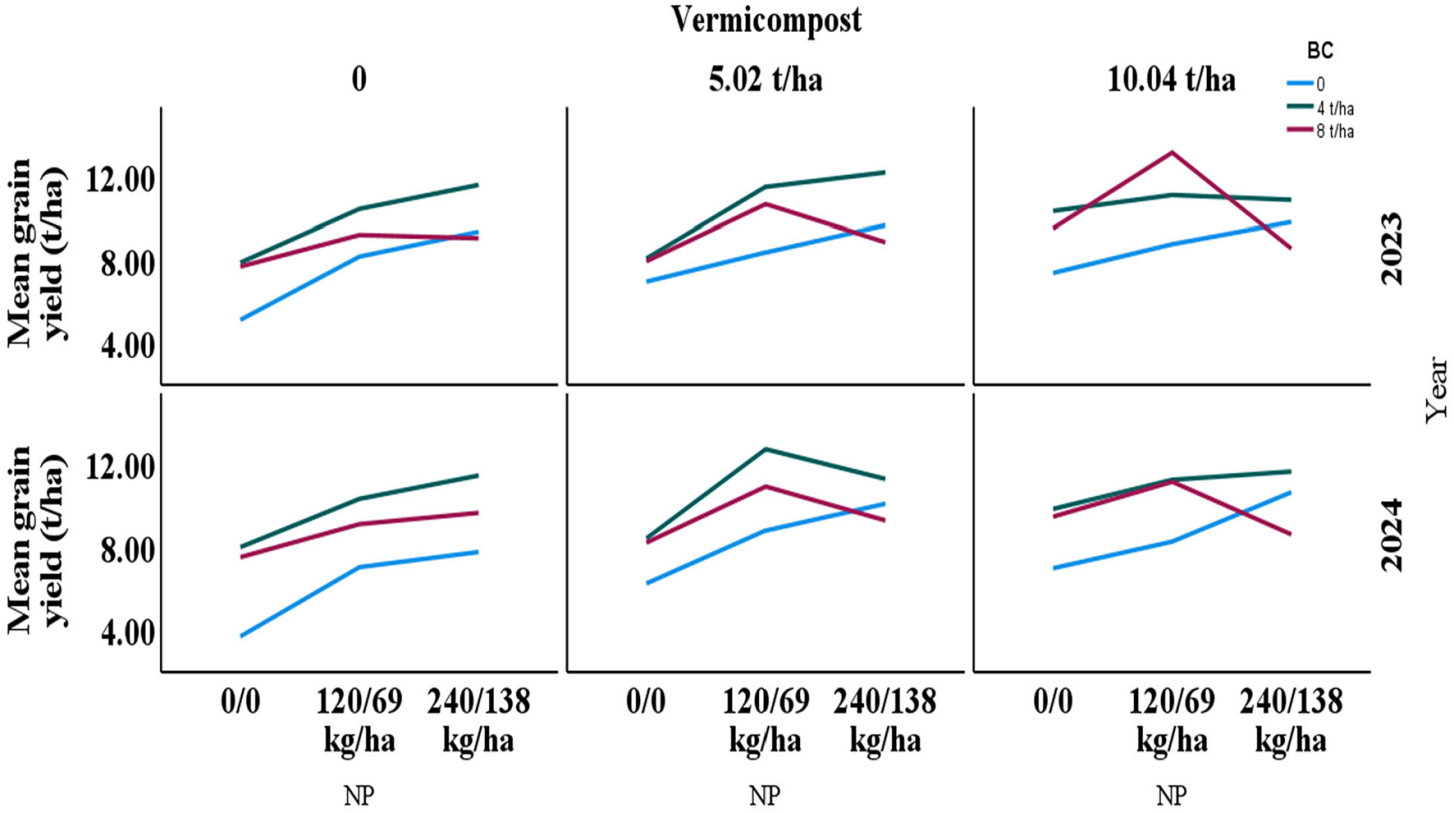
Three-way interaction of N/P₂O₅ × BC × VC on maize grain yield, based on least square means from the proc mixed model.

The ANOVA results showed the grain yield was highly significantly (*P* < 0.0001) affected by the N/P_2_O_5_, BC, VC, year, two-way and three-way interactions, whereas the interaction effects of N/P_2_O_5_ × Year was not significant (*P* ≥ 0.05) (Table 7 and Fig. 3). All treatments were conducted under a uniform lime application, which served as the baseline for assessing additional treatment effects. In 2023/24, the highest grain yield was observed in T24 (13.15 t ha^−1^), T8 (12.19 t ha^−1^), and T14 (11.59 t ha^−1^), whereas the lowest grain yield occurred in the limed control. In 2024, T14 produced the highest grain yield (12.68 t ha^−1^), followed by T26 (11.61 t ha^−1^), with the lowest yield recorded in T1 (3.67 t ha^−1^) under the limed baseline. Across the two-year experiment, sole applications of inorganic nutrients produced lower grain yield compared with combined organic–inorganic treatments (Supplementary Table S2, Fig. 4, and Fig. 5). The mean grain yields over both years was slightly higher in 2023 (9.34 t ha^−1^) than in 2024 (9.17 t ha^−1^). On average, the highest grain yield (12.13 t ha^−1^) was observed in T24 (120/69 kg N/P_2_O_5_ ha^−1^ + 8 t BC ha^−1^ + 10.04 t VC ha^−1^), which was statistically on par with T14 (12.09 t ha^−1^) and T17 (11.72 t ha^−1^). The lowest GY (4.40 t ha^−1^) was recorded in T1, which received uniform lime amendments (Fig. 4).

Overall, soil chemical quality significantly enhanced grain yield during the trial. Treatments combining inorganic and organic amendments produced the greatest gains: T24 (175.7%), T14 (174.8%), T17 (166.36%), T7 (240/138 kg N/P₂O₅ ha^−1^; 4.71%), T19 (8 t BC ha^−1^; 59.82%), and T3 (10.04 t VC ha^−1^; 69.41%), relative to the limed baseline (Fig. 4). The maximum absolute increase in yield over the control was 7.73 t ha^−1^. Sole applications of 240/138 kg N/P₂O₅ ha^−1^, 8 t BC ha^−1^, and 10.04 t VC ha^−1^ resulted in yield gains of 4.15, 2.76, and 3.39 t ha^−1^, respectively.

The combined application of inorganic and organic (T14; N/P₂O₅, BC, and VC) produced superior yields, with mean increases of 41.4%, 59.29%, and 68.85%, respectively, compared with their maximum individual applications. Pearson correlation analysis showed t that grain yield was positively and highly significantly correlated with SOC (r = 0.693**), SOM (r = 0.694**), TN (r = 0.688**), avP (r = 0.649**) and soil pH (r = 0.529**), while being highly significantly and negatively correlated with Exc. Ac (r = -0.511**) and Exc. H (= -0.577**). Grain yield was not significantly correlated with Exc. Al (r = 0.284^ ns^), Table 8.

### ***Pearson correlation coefficients (r) among selected soil chemical properties and yield of maize influenced by N/P***_***2***_***O***_***5***_***, BC, and VC levels***

The relationships between the soil chemical properties and crop parameters are illustrated in Table 9. Results showed in Table 10 show the outcomes of Bartlett’s test of sphericity and the Kaiser–Meyer–Olkin (KMO) measure of sampling adequacy. Our KMO value is between 0.6 and 0.7, which is considered acceptable. Bartlett’s test (*P* < 0.0001, < 0.05) revealed that the correlation matrix is not identical and that factor analysis is appropriate.

### KMO analysis of soil properties and maize parameters

The KMO value was 0.663 (Table 9), which is just above the minimum acceptable threshold of 0.50, indicating that the dataset is suitable for factor analysis^57–60^. Similarly, Williams et al.^60^ reported that KMO values above 0.50 are considered acceptable for factor analysis. Bartlett’s test of sphericity tests the null hypothesis that the correlation matrix is an identity matrix (variables are unrelated). A significant result (*P* < 0.05) indicates that the variables are sufficiently correlated for factor analysis to be appropriate^60^. The actual P-value from our analysis (< 0.001) is reported in Table 9, which is consistent with the corrected threshold.

### Proc mixed model results

The proc mixed analysis showed that all main effects (NP, BC, VC) significantly influenced maize grain yield (fixed effects; Table 10, Fig. 4). The three-way interaction NP × BC × VC was significant, indicating non-additive combined effects, whereas some two-way interactions were not significant, suggesting weaker interactions. Among random effects, year variance was 0.8123 t2 ha^−2^, reflecting moderate variation between the two growing seasons, and replication (within year) variance was 0.4578 t2 ha^−2^, indicating variability among replications. The residual (experimental error) was 1.24 t2 ha^−2^, representing unexplained variation. Denominator degrees of freedom (df) were constant at 8, as the fully balanced design (all factor combinations with equal replications per year) allowed SAS to apply the Satterthwaite approximation, yielding identical df for all fixed effects. Random effects are reported only as variance components without F- or P-values (Table 10).

### Evaluation of multiple linear regression models

**Fig. 6:**
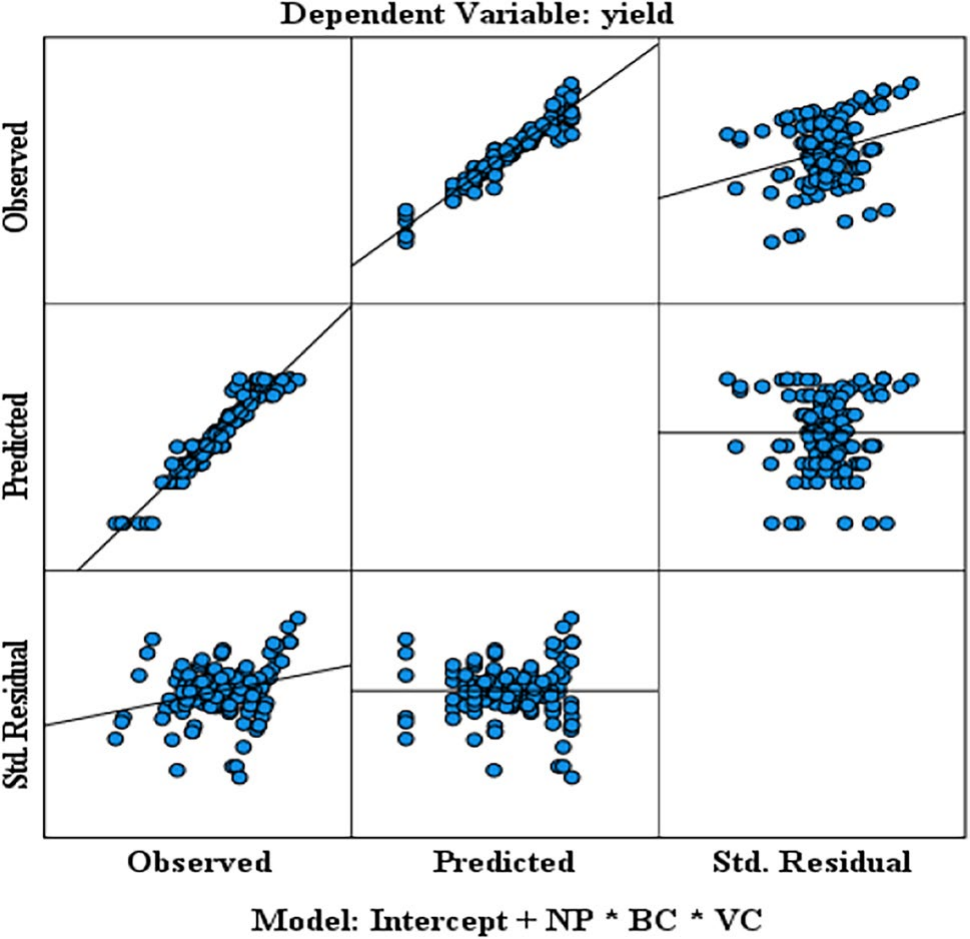
Scatterplot matrix showing relationships among observed grain yield, predicted grain yield, and standardized residuals for the fitted multiple linear regression model (Intercept + NP, N/P₂O₅ + BC × VC).

Variation in soil properties and maize yield was generated using different rates of N/P₂O₅, BC, and VC to enable reliable model evaluation. Treatment levels were as predictors and variables were selected based on Pearson correlation (P < 0.05; r > 0.40). Multicollinearity was minimized by excluding variables with variance inflation factors (VIF ≥ 10). Most soil chemical properties were positively associated with grain yield, whereas exchangeable H, Al, and total acidity showed negative relationships. However, exchangeable Al was not significantly correlated with yield (P > 0.05). The model achieved an RMSE of 0.92 t ha^−1^ and an R^2^ of 0.655, explaining 65.5% of the variation in maize yield. These results indicate the predictive relevance of selected soil and crop variables and highlight the importance of accurate input data for improving model performance, which is further supported by the close agreement between observed and predicted values and the random distribution of residuals (Fig. 6). The strong linear agreement between observed and predicted values indicates good predictive performance, while the random dispersion of standardized residuals around zero suggests no evident violation of model assumptions or systematic bias.

### Equations and coefficients for the MLR models

The development of the multiple linear regression model (MLRM) involved estimating coefficients that best describe the relationships between selected predictors and maize grain yield (Table 11). Predictor variables included N/P₂O₅ rates, BC, VC, soil pH, TN, avP, and SOC. These variables satisfied model assumptions, exhibiting acceptable multicollinearity (VIF < 10)^61^ and moderate to strong correlations with grain yield (r > 0.40)^62^. The fitted model expresses grain yield as a function of these predictors, with each coefficient representing the expected change in yield per unit increase in the corresponding variable, holding others constant. The intercept (β₀ = − 20.79) represents the theoretical baseline yield when all predictors are zero.

Among the variables, TN exhibited the strongest positive effect on yield, followed by VC and BC. Specifically, a one-unit increase in N/P₂O₅ rate (0/0 to 120/69 to 240/138) was associated with a 0.535 t ha^−1^ increase in yield (*P* ≥ 0.05). Similarly, one-unit increases in BC (0–8 t ha^−1^) and VC resulted in yield increases of 0.879 t ha^−1^ (*P* ≥ 0.01) and 2.84 t ha^−1^ (*P* ≤ 0.05), respectively. Soil TN showed a substantial positive effect, with a 12.15 t ha^−1^ increase per unit (*P* ≥ 0.05), while avP and SOC contributed increases of 0.017 and 2.301 t ha^−1^, respectively (*P* ≥ 0.05). Detailed model coefficients are presented in Table 12. For the LR model used in this study, the equation was:

$$Yield=-20.79+0.54\times \mathrm{N}\mathrm{P} \mathrm{r}\mathrm{a}\mathrm{t}\mathrm{e}\mathrm{s} +0.88\times \mathrm{B}\mathrm{C} \mathrm{r}\mathrm{a}\mathrm{t}\mathrm{e}\mathrm{s}+0.09\times \mathrm{V}\mathrm{C}+2.85\times \mathrm{p}\mathrm{H}+12.145\times \mathrm{T}\mathrm{N} +0.02\times \mathrm{a}\mathrm{v}\mathrm{P}+2.30\times \mathrm{S}\mathrm{O}\mathrm{C}+\upvarepsilon$$

Abbreviations: N, nitrogen rate (kg ha^−1^); P_2_O_5_, phosphorus rate (kg ha^−1^); BC, biochar rate (t ha^−1^); VC, vermicompost rate (t ha^−1^); pH, soil pH; SOC, soil organic carbon (%); TN, soil total nitrogen (%); avP, available soil phosphorus (mg kg^−1^); ε, Error term (variation not explained by predictors).

### Model validations and comparison

**Fig. 7:**
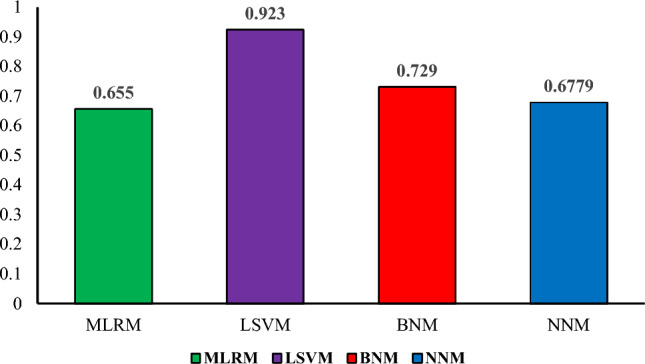
Model performance evaluation based on coefficient of determination (R^2^). MLEM, Multiple Linear Regression Model; LSVM, Linear Support Vector Machine; BNM, Bayesian Network Model; NNM, Neural Network Model.

Table 11 and Fig. 5 showed that the MLR model precisely predicted potential grain yield from actual yield, soil, and grain yield, with low error values (MSE = 0 t ha^−1^, close to zero which is desirable; MPE = – 1.28%, slight under estimate). Among the four models used linear victim support model was found the best model with R^2^ of 0 0.92 (Fig. 7).

### Partial budget analysis

Partial budget analysis results are presented in Table 14. Sole applications of high N/P₂O₅ nutrient rates (e.g., 240/138 kg ha^−1^), 10.04 t VC ha^−1^, and 8 t BC ha^−1^ were not economically viable despite producing higher yields, due to their dominance and high cost. For example, 240/138 kg N/P₂O₅ ha^−1^ yielded a net benefit of 193,957.95 ETB ha^−1^ but was economically dominated. In contrast, integrated treatments showed significantly higher profitability. Net benefits followed the order: T14 > T16 > T13 > T17 > T24 > T15 > T12 > T23 > T10. Only treatments T10, T13, and T14 achieved a marginal rate of return (MRR) above the minimum acceptable threshold of 100%. The most profitable treatment was 120/69 kg N/P₂O₅ ha^−1^ + 5.02 t VC ha^−1^ + 4 t BC ha^−1^ (T14), which resulted in a net benefit of 289,124 ETB ha^−1^ and an MRR of 149.07%, indicating a return of 1.49 ETB for every 1 ETB invested. Treatments T13 and T10 also surpassed the 100% MRR threshold, confirming their profitability under local price conditions. Overall, sole applications of inorganic N/P₂O₅, BC, or VC were less profitable compared to integrated treatments.

### Sensitivity analysis

To assess the reliability of economic recommendations, underprice variability, we conducted a simple sensitivity analysis for the most profitable treatment (T14) by varying key parameters ± 20% (Table 13). Under all scenarios tested, T14 maintained MRR above the 100% minimum acceptable threshold, indicating vigorous profitability even with adverse price fluctuations. The treatment remained profitable with grain price decreases up to 30% (MRR = 94.3%) but fell below 100% at 35% price reduction. These findings suggest that the integrated treatment provides a risk-averse investment for smallholder farmers.

## Discussion

### Effect of organic and inorganic amendments on soil acidity

Soil pH was strongly influenced by the integrated application of BC, VC, and inorganic N/P₂O₅, with the highest pH observed under combined BC and VC application (8 t ha^−1^ BC + 10.04 t ha^−1^ VC) and the lowest under sole inorganic fertilization. The uniform lime application across all plots may have interacted with treatments, limiting the ability to isolate the effects of the organic amendments alone.

Application of BC and VC also significantly reduced exchangeable acidity (Exc. Ac), exchangeable hydrogen (Exc. H⁺), and exchangeable aluminum (Exc. Al) compared with the control and sole mineral fertilization. The maximum BC + VC treatment (T21) decreased Exc. Al by approximately 71.13% and Exc. H⁺ by 71.1% relative to high N/P₂O₅ application alone (T7). A consistent decline in Exc. Ac was observed with increasing BC and VC rates, indicating the ameliorating effect of these organic inputs, whereas inorganic fertilization alone increased Exc. Ac, confirming that repeated use of ammonium-based fertilizers exacerbates soil acidification.

The reduction in Exc. Ac is likely attributable to the high alkalinity and cation content of BC and VC, which displace H⁺ and Al^3^⁺ from exchange sites and promote precipitation of Al as Al(OH)₃. The observed pH increase under BC and VC is attributable to their alkalinity, supply of basic cations, and enhancement of cation exchange capacity, consistent with the buffering effects of organic amendments in acidic soils. The combined effect of lime and biochar likely involves synergistic mechanisms: lime provides a rapid pH increase via CaCO₃ dissolution, while biochar contributes longer-term buffering through its alkaline functional groups and base cations. This synergy may explain why the 8 t BC ha^−1^ + 10.04 t VC ha^−1^ treatment reached a pH of 5.82 despite receiving only 25% of the recommended lime rate. In contrast, high mineral N application reduced soil pH, likely due to acidification from nitrification and leaching of exchangeable bases. Positive correlations between pH, SOM, and available P highlight the role of organic inputs in stabilizing soil chemical properties. Additionally, the increase in exchangeable acidity (Exc. Ac) and exchangeable aluminum (Exc. Al) under sole N application aligns with widely reported trends in acidic soils, emphasizing the potential risks of exclusive mineral N fertilization on soil chemical stability.

The reduction in exchangeable Al^3^⁺ (up to 71%) under combined treatments can be attributed to multiple interacting mechanisms:

1. *pH-mediated precipitation* Soil pH increased from 4.75–4.87 to 5.62–5.82 under integrated treatments, causing soluble Al^3^⁺ to precipitate progressively as Al (OH)₃, which is non-toxic to plants^5^. Aluminum toxicity in maize is typically associated with acidic soils (pH < 5.0), where soluble Al^3^⁺ concentrations increase sharply and impair root growth and nutrient uptake.
2. *Organic complexation* Biochar and vermicompost contain oxygen-containing functional groups (carboxyl, phenolic hydroxyl, carbonyl) that form stable organo-aluminum complexes with Al^3^⁺ (Lemann and Joseph, 2015). These complexes are not exchangeable with 1 M KCl and therefore do not appear in exchangeable Al measurements, while remaining not harmful to plants.
3. *Cation competition* Cation competition likely contributed to the reduction in exchangeable Al^3^⁺: calcium from lime and base cations (Ca^2^⁺, Mg^2^⁺, K⁺) supplied by BC and VC increased base saturation and displaced Al^3^⁺ from exchange sites as soil pH increased, lowering exchangeable Al^3^⁺^62,63^.
4. *Specific adsorption* Biochar’s aromatic carbon structure and oxygen-containing functional groups can adsorb Al^3^⁺ through cation–π, electrostatic, and surface complexation mechanisms, contributing to reduced exchangeable Al^3^⁺ in acidic soils^63,64^.

The relative importance of these mechanisms likely shifts over time: liming effects dominate initially, while organic complexation and surface adsorption become increasingly important as biochar ages and vermicompost decomposes. Similar studies in acidic soils of western Ethiopia^5^ and Kenya^63^ reported comparable reductions in exchangeable Al with integrated organic–inorganic amendments.

The observed effects of organic amendments in this study corroborate earlier findings that materials such as BC and VC act as liming agents, increasing soil pH, supplying essential base cations, and reducing aluminum (Al3⁺) toxicity. Previous reports indicate that high BC application can raise pH by 0.6–2.0 units, supporting our observation that the combined use of BC and VC effectively mitigates acidity in strongly acidic soils. The reduction in exchangeable Al3⁺ is particularly agronomically important, as it lowers phosphorus fixation and improves overall nutrient availability.

Although the initial soil condition indicated potential aluminum toxicity, the integrated application of BC, VC, and N–P fertilizer substantially reduced soil acidity and exchangeable Al during the experimental period. Consequently, the direct inhibitory effect of Exc. Al on maize grain yield may have been mitigated through improved soil pH, enhanced nutrient availability, and Al detoxification mechanisms associated with the applied amendments. In addition, grain yield appeared to be more strongly influenced by other improved soil properties such as soil pH, available phosphorus, organic carbon, and total nitrogen than by residual Exc. Al levels alone. Therefore, despite elevated baseline Exc. Al values, the observed correlation between Exc. Al and grain yield remained statistically non-significant (r = 0.284, ns).

These results align with previous studies indicate that VC, compost, and BC can mitigate soil acidity and raise pH in acidic soils. For instance, Agegnehu et al.^7^ reported significant pH increases when BC was applied alongside inorganic fertilizers, while Odoemelam and Ajunwa^65^ observed increases from pH 4.68 to 7.19 following organic amendments. Likewise, Emamu and Wakgari^66^,Gadisa and Wakgari^23^, and Bekele et al.^5^ reported that integrating VC with mineral fertilizers or lime effectively raised soil pH compared with control or fertilizer-only treatments. These studies indicate that combining BC and VC with moderate inorganic nutrients substantially reduces Exc. Ac and improves soil chemical properties relative to mineral fertilization alone.

Overall, integrating BC and VC with moderate inorganic nutrient inputs provides an effective strategy to reduce exchangeable acidity, alleviate Al3⁺ toxicity, and enhance chemical fertility in acidic Nitisols. The apparent discrepancy between strongly acidic pH values and moderate exchangeable acidity arises because exchangeable acidity quantifies only Al3⁺ and H⁺ on exchange sites, whereas pH reflects the concentration of H⁺ in the soil solution. In highly weathered soils such as Nitisols, substantial acidity may persist as exchangeable Al3⁺ even when solution pH appears moderate^1^. Thus, the coexistence of low pH and elevated Al3⁺ confirms that soil acidity remains a significant constraint to crop productivity.

### Impact of organic and inorganic amendments on soil fertility parameters

Soil total nitrogen (TN) was significantly enhanced by the combined application of inorganic N/P₂O₅ nutrients with biochar (BC) and vermicompost (VC) (Table 5). The highest TN (0.322%) was recorded under 120/69 kg N/P₂O₅ ha^−1^ + 4 t BC ha^−1^ + 5.02 t VC ha^−1^, exceeding values observed under single mineral, BC, or VC treatments. The mean TN (0.286%) was higher than the 0.21% reported by Amare et al.^14^ during three years of continuous maize with mineral and VC fertilization, suggesting that the synergistic effects of BC and VC improved N retention and cycling. This improvement is likely due to both direct N supply from fertilizers and indirect effects of organic amendments. Together, these effects, along with reduced leaching losses and enhanced microbial mineralization, contributed to higher TN.

A strong positive correlation between soil TN and SOC (r = 0.866**) indicates that organic matter accumulation substantially contributed to soil N enrichment. In contrast, the control treatment had the lowest TN, reflecting continuous N mining by crops without replenishment. The observed shift in TN from low to optimum ranges with increasing rates of VC and BC supports the role of organic amendments in improving soil fertility. Comparable findings have been reported elsewhere, where VC and integrated nutrient management consistently increased TN relative to chemical fertilization alone^5,67,68^. Previous studies also reported higher TN under combined organic and inorganic fertilization than with single inputs^66,69^.

The improved N availability can be attributed to:

(i) provision of direct N from inorganic N/P₂O₅;
(ii) reduction of N leaching due to biochar’s high adsorption capacity;
(iii) gradual release of N from VC matching crop uptake patterns;
(iv) enhancement of biological N fixation by free-living organisms stimulated by organic amendments; and.
(v) improved root exploration, allowing greater N acquisition from the soil volume^5,67,68^.

Soil available phosphorus (avP) increased consistently with higher application rates of inorganic N/P₂O₅, BC, and VC (Table 6). Compared with the control, avP increased markedly under 120/69 and 240/138 kg N/P₂O₅ ha^−1^, 4–8 t BC ha^−1^, and 5.02–10.04 t VC ha^−1^. The combined application of these amendments resulted in the highest avP (48.38 mg kg^−1^), whereas the control recorded the lowest value (13.29 mg kg^−1^). These improvements can be attributed to the combined supply of P from NPSB fertilizer and additional P release from BC and VC, as supported by strong correlations between avP and SOC (r = 0.729**) and avP and TN (r = 0.874***).

The increase in P availability under integrated nutrient application is likely associated with reduced P fixation in acidic soils, where applied P typically precipitates with Al3⁺ and Fe3⁺. Although treatments significantly affected soil pH, the lack of correlation with avP may reflect temporal variability, P fixation processes, and interactions with organic amendments. Organic amendments can enhance mineralization, provide organic ligands that complex with Al and Fe, and improve soil pH buffering, thereby increasing the labile P pool. The mean avP across treatments was 32.35 mg kg^−1^ (Mehlich-II), highlighting the effectiveness of integrated nutrient management.

The mechanisms underlying enhanced P availability in integrated treatments include:

(i) provision of direct P from NPSB fertilizer and pH amelioration by biochar;
(ii) reduction of P fixation through complexation of Al3⁺ and Fe3⁺ by organic ligands;
(iii) increase in soil pH, shifting P towards plant-available H₂PO₄^−^ forms;
(iv) competition of organic anions with phosphate for sorption sites; and
(v) stimulation of microbial activity producing phosphatases that mineralize organic P^63^.

Similarly, Singh et al.^70^,Inal et al.^71^, and Habtamu et al.^72^ reported higher P availability under combined vermicompost and mineral fertilizer than with fertilizer alone. The observed improvements may also result from direct P contributions from BC and enhanced microbial activity facilitating mineralization and organic acid production. Optimal P availability occurs in moderately acidic soils (pH 5.5–6.0), where H₂PO₄^−^ predominates, suggesting that BC and VC contribute both to nutrient supply and pH buffering, thereby enhancing P availability.

Soil organic carbon (SOC) was significantly increased by the combined application of inorganic N/P₂O₅ nutrients with BC and VC, reaching 2.65% compared with 1.55% in the control (Table 6). The increase observed from the initial SOC (2.11%) and the unfertilized control underscores the importance of integrated nutrient management in sustaining soil quality. A positive correlation between SOC and soil pH (r = 0.404) suggests that pH improvement following BC and VC application alleviated acidity-related stress, supporting microbial activity and stabilizing organic matter.

Biochar contributed primarily through its role as a soil amendment rather than as a direct nutrient source. Its effects included pH amelioration, increased cation exchange capacity (CEC), and retention of nutrients from other sources. While BC contains some nutrients (1.11% N, 46.5 mg kg^−1^ P), only a small fraction (< 5% of N and P) is plant-available in the first year due to the recalcitrant nature of biochar^29^. Vermicompost, in contrast, supplied readily available nutrients, particularly N, which, combined with BC, resulted in synergistic improvements in SOC and soil fertility.

The highest soil organic matter (SOM, 4.62%) was recorded under 120/69 kg N/P₂O₅ ha^−1^ + 8 t BC ha^−1^ + 5.02 t VC ha^−1^, following a trend similar to SOC. The positive correlation between total N (TN) and SOM (r = 0.865**, *P* ≤ 0.001) indicates that organic amendments enhance nutrient cycling and microbial activity, contributing to carbon stabilization^73^.

The findings of this study confirm that organic amendments, alone or combined with fertilizers, increase SOC and SOM through organic substrate addition, improved residue decomposition, and stimulation of microbial processes^7,74,75^. Increases in SOC can also be attributed to the stability of biochar-derived carbon, higher root biomass input, and improved soil aggregation. Vermicompost is particularly rich in N^76^, and its integration with BC further enhances SOM and TN.

Overall, the integrated application of BC, VC, and inorganic nutrients effectively improves SOC and SOM, enhances microbial activity, and stabilizes soil chemical properties, offering a sustainable strategy to improve soil fertility and resilience in acidic Nitisols.

### Influence of organic amendments on microbial activity and nutrient synchronization

Although microbial parameters were not directly measured in this study, the observed improvements in soil chemical properties suggest enhanced microbial activity under integrated treatments. Several mechanisms likely contributed:

1. *Relief from acidity stress* Soil microorganisms are sensitive to low pH and high Al^3^⁺ concentrations^13^. Increasing soil pH from 4.8 to above 5.5 likely alleviated this stress, supporting microbial population recovery. Previous studies have reported 2–threefold increases in microbial biomass when acidic soils are limed to pH > 5.5.
2. *Carbon substrate supply* Vermicompost provides readily available carbon compounds such as sugars, amino acids, and organic acids, which fuel microbial metabolism^77^. Biochar, while more recalcitrant, offers porous microhabitats that protect microorganisms from predation and desiccation^73^. Enhanced availability of N and P (Table 6) further supports microbial growth, and strong correlations between SOC, TN, and avP (r > 0.7) suggest tightly coupled cycling of carbon, nitrogen, and phosphorus through microbial biomass.
3. *Enzyme stimulation* Organic amendments have been shown to increase the activities of key soil enzymes, including dehydrogenase, urease, and phosphatase, which mediate nutrient mineralization and serve as sensitive indicators of biological soil quality^78^. Future studies are recommended to directly quantify microbial biomass, community composition (e.g., via Phospholipid Fatty Acid Analysis or metagenomics), and enzyme activities to confirm these mechanisms.

### Nutrient synchronization

Nutrient synchronization, the temporal alignment of nutrient release from amendments with crop uptake, is a critical factor in the success of integrated nutrient management^79^. In this study, several factors likely contributed to improved synchronization:

1. *Differential release rates* Inorganic fertilizers supplied immediately available N and P, meeting early crop demand. Vermicompost released nutrients gradually through microbial mineralization, with peak N availability typically 4–8 weeks after application, coinciding with maize’s rapid vegetative growth phase^77^. Biochar contributed minimal directly available N but retained nutrients through adsorption, reducing leaching losses^13^.
2. *Split N application* Urea was applied in three splits (28.8% at planting, the remainder at knee-height and tasseling), aligning N supply with peak crop uptake. This practice has been shown to improve N use efficiency in maize by 20–30% compared to single applications^15,24^.
3. *Reduced leaching* Biochar’s high adsorption capacity for NH₄⁺ and NO₃^−^ likely reduced N leaching during periods of high rainfall, particularly in August when monthly rainfall exceeded 400 mm (Fig. 2). Studies in tropical sandy soils report 30–50% reductions in N leaching with biochar application^63^.
4. *Priming effects* Addition of fresh organic matter from vermicompost may have stimulated microbial mineralization of native soil organic matter and even biochar surfaces over time^28^, providing additional nutrients during the growing season.

The yield benefits observed under integrated treatments (175% increase over control) likely reflect not only greater total nutrient supply but also improved temporal alignment of nutrient availability with crop demand, reducing losses and enhancing uptake efficiency.

### Treatment effects on maize grain yield

The grain yields obtained in this study (up to 12.13 t ha^−1^) substantially exceed the national average maize yield in Ethiopia, which is approximately 4.1 t ha^−1^ based on recent USDA estimates^80^ and the regional averages for the Amhara highlands (3 t ha^−1^)^36^. These high yields are consistent with the documented potential of the BH-661 hybrid, which has reported research yields of 9.5–12 t ha^−1^ under optimal management (Amhara Regional State Agricultural Extension Agronomy Package, 2022). The relatively higher CV value (20%) for grain yield was mainly attributed to seasonal variation between the two experimental years. Grain yield is highly influenced by environmental factors such as rainfall distribution, temperature, and other climatic conditions, which differed between years and increased variability among observations. Despite this variability, the CV remained within an acceptable range (10–20%) for field experiments involving multi-year yield data. The observed yields reflect the combined influence of genetic potential, precise agronomic management, integrated soil fertility practices, and favorable environmental conditions. This could be;

### Genetic potential

The use of BH-661, a high-yielding hybrid adapted to 1600–2200 m.a.s.l., provided a strong foundation for productivity. Certified seeds ensured genetic purity and high germination rates, enabling the crop to express its full yield potential. Previous studies have documented farmer-level yields of 6.5–8.5 t ha^−1^ under good management and research station yields to more than 12 t ha^−1^^14,18^. Our results (up to 13.15 t ha^−1^) represent the upper end of the hybrid’s potential, reflecting optimal management and favorable site conditions. However, the highest grain yield was not economically optimal.

### Optimal agronomic management

Plant populations were carefully maintained at 90,909 plants ha^−1^ through precise spacing (0.55 m × 0.20 m) and timely thinning. Split nitrogen application (28.8% at planting, remainder at knee-height and tasseling) synchronized nutrient supply with maize phenology, reducing losses from leaching and volatilization^19^. Two rounds of hand weeding at critical growth stages (25 and 45 days after planting) minimized nutrient competition from weeds.

### Integrated soil fertility management

Combining organic amendments with reduced rates of inorganic fertilizers addressed both nutrient supply and chemical constraints. Biochar contributed liming effects and improved cation exchange capacity, while vermicompost provided readily available nutrients and stimulated microbial activity. Uniform lime application mitigated aluminum toxicity, creating favorable root zone conditions. The use of maize cob biochar represents a sustainable and circular approach by converting crop residues that are often burned into a beneficial soil amendment, thereby reducing waste and greenhouse gas emissions^73,81^.

### Environmental conditions

Sub-humid climate with adequate rainfall (1,654 mm annually) during both cropping seasons (Fig. 2), combined with proper drainage via bunds and ditches, maintained optimal soil moisture without waterlogging stress. These conditions, together with high-quality organic amendments, facilitated nutrient availability and uptake. The economically optimal treatment (T14: 120/69 kg N/P₂O₅ ha^−1^ + 4 t BC ha^−1^ + 5.02 t VC ha^−1^) produced 12.09 t ha^−1^ with a marginal rate of return of 149%, demonstrating that high yields can be economically viable when inorganic fertilizers are partially substituted with locally available organic inputs. Adoption by smallholder farmers depends on factors including labor for biochar production (15–20 person-days per tonne), skills for vermicomposting, upfront costs for fertilizers, and risk aversion. Participatory extension approaches in Ethiopia, however, have shown that with adequate training, farmers can achieve yields of 7–9 t ha^−1^ using improved practices^24,36^.

### Yield improvements and mechanisms

The 175.77% yield increase over the control observed in this study exceeds the 42% improvement reported by Bekele et al.^5^ under lime plus vermicompost, which may be attributed to the synergistic effects of biochar-induced liming and vermicompost-derived nutrient supply; however, variability in agroecosystem conditions should be taken into account when interpreting these differences. Similar studies report yield increases of 40–70% with biochar-compost combinations^7^ or significant gains with biochar plus NPS fertilizer^21^. Positive and significant correlations between grain yield and soil properties pH (r = 0.529**), TN (r = 0.688**), SOM (r = 0.694**), SOC (r = 0.693**), and available P (r = 0.649**) underscore the role of improved soil fertility in driving yield. Negative correlations with exchangeable H⁺ and Al^3^⁺ highlight the detrimental effects of soil acidity on crop performance.

### Comparison with previous studies

Globally, integrated soil fertility management approaches have consistently increased maize yields. For example, in Northern Ghana, the combined application of biochar, compost, and mineral fertilizer increased maize yield by more than 100% compared to the control^82^. Long-term trials in Kenya further indicate that biochar application combined with mineral fertilizer enhances and sustains maize yield over time^83^, which is consistent with the findings of the present study. Similarly, in Ethiopia, integrated application of compost and mineral fertilizer significantly improved maize yield compared to sole inputs^84^. Ashenafi et al.^24^ achieved 8–10 t ha^−1^ with farmyard manure plus fertilizers. Likewise, Chimdessa^84^ in western Oromia reported significant yield improvements with vermicompost plus NPS fertilizer.

Overall, these results showed that integrated soil fertility management using BC, VC, and reduced inorganic N/P₂O₅ maximizes maize grain yield by improving soil chemical properties, promoting nutrient synchrony with crop demand, and mitigating acidity constraints. While experimental yields exceed typical smallholder harvests, adoption of these practices can substantially increase maize productivity and sustainability in the Ethiopian highlands.

### Cross-validated datasets and relationships between soil properties and crop parameters

Cross-validation using a 20:80 train–test split showed a moderate correlation in the training dataset (r = 0.541, *P* = 0.210) and a strong, significant correlation in the validation dataset (r = 0.752, P < 0.01), indicating good predictive performance despite limited training data.

Grain yield was positively correlated with soil organic matter (SOM; r = 0.694**), soil organic carbon (SOC; r = 0.693**), total nitrogen (TN; r = 0.688**), available phosphorus (avP; r = 0.649**), and pH (r = 0.529**), but negatively correlated with exchangeable acidity (r = − 0.511*) and exchangeable hydrogen (r = − 0.577**), while exchangeable aluminum had no significant effect. Soil pH was strongly negatively associated with exchangeable hydrogen (r = − 0.780**) and exchangeable acidity (r = − 0.945**), indicating that improved pH reduces acidity constraints.

These findings align with previous studies in Ethiopian highlands, where grain yield was positively associated with SOM and soil pH and negatively associated with exchangeable acidity and aluminum^5,85–87^.

### Model performance and comparison

Different models have been applied to predict crop yield using soil and management variables^88–90^. In this study, four models were evaluated: Multiple Linear Regression (MLRM), Linear Support Vector Machine (LSVM), Bayesian Network Model (BNM), and Neural Network Model (NNM), representing linear, nonlinear, probabilistic, and complex learning approaches, respectively.

Model performance metrics (MAPE = 9.78%, RMSE = 0.92 t ha^−1^, MAE = 0.92 t ha^−1^, R^2^ = 0.655) indicate that MLRM predicted maize yield with good accuracy, with MAPE < 10% reflecting reliable performance^91^. Among the models, LSVM achieved the highest accuracy (R^2^ = 0.923), effectively capturing complex interactions, whereas BNM (R^2^ = 0.729) and NNM (R^2^ = 0.678) performed less well. However, NNM identified soil TN, pH, SOC, and avP as the most influential predictors, while inorganic N had minimal contribution.

These findings emphasize the critical role of soil fertility improvements via BC and VC^8,16,17,90^. Overall, LSVM emerged as the most reliable and interpretable model for predicting maize yield under integrated nutrient management in acidic Nitisols.

### ***Cost benefit analysis for the combined application of N/P***_***2***_***O***_***5***_***, BC and VC***

The application of integrated inputs in T14 increased the net benefits by 49.06%, 76.83%, and 46.08% over the recommended sole applications of T7, T3, and T19, respectively. These findings support the use of localized organic inputs such as maize cob BC and VC to reduce the reliance on expensive synthetic fertilizers. Vermicompost, for example, can substitute up to 23.9 kg of nitrogen per tonne, helping resource-poor farmers improve productivity affordably in the Burie district.

Our study is consistent with that of Tufa et al.^22^, who reported profitability rates of 8 t ha^−1^ BC and 50 kg NPS (nitrogen, phosphorus, sulfur) ha^−1^in the Guto Gida district. Moreover^92^, reported the greatest net benefit when 10 t ha^−1^ FYM (farmyard manure) and 69 kg N ha^−1^ were used. Similar integrated benefits were reported by Woubshet et al.^6^,Ejigu et al.^83^ and Ashenafi et al.^25^. An earlier study by Khuong et al.^93^ also emphasized the role of site-specific NPK (nitrogen, phosphorus, potassium) management combined with organic fertilizers in enhancing marginal returns. Thus, the ISFM, especially T14, which is followed by T26, is recommended for maize growers in acidic soils of Burie district. These findings indicate that the integrated application of BC, VC, and inorganic N/P_2_O_5_ nutrients can improve maize grain yield and soil chemical quality under acidic conditions.

The economic results have important implications for smallholder farmers. Treatment T14 (120/69 kg N/P₂O₅ ha^−1^ + 4 t BC ha^−1^ + 5.02 t VC ha^−1^) achieved the highest net benefit (289,124 ETB ha^−1^) and MRR (149%) while using 50% less inorganic fertilizer than the full recommended rate. This suggests that farmers could potentially reduce cash expenditures on fertilizers by investing labor in producing biochar and vermicompost from locally available materials. The sensitivity analysis indicates that even with unfavorable price changes (+ 20% input costs or -20% grain prices), T14 remains profitable above the 100% MRR threshold. However, the labor requirements for organic amendment production (estimated at 25–30 person-days ha^−1^ for biochar + 15–20 person-days ha^−1^ for vermicompost) may be constraining for labor-limited households.

## Conclusions

This two-year, single-location study indicate that integrating BC and VC with reduced inorganic fertilizer, against a background of lime application, can significantly enhance soil chemical quality and maize productivity in acidic Nitisols of the Burie district, Ethiopia. Combined applications improved key soil chemical properties, with pH increasing by up to 0.95 units, SOC by 0.53%, TN by 0.07%, available P by 27.5 mg kg^−1^, and Exc. Ac decreasing by up to 1.24 cmol₍c₎ kg^−1^, while mitigating aluminum toxicity. These improvements translated to substantial yield gains, with the optimal treatment (120/69 kg N/P₂O₅ ha^−1^ + 8 t BC ha^−1^ + 10.04 t VC ha^−1^) increasing maize grain yield by 175.7% compared with the unfertilized control. A treatment combining 50% of the recommended inorganic N/P_2_O_5_ with 4 t BC ha⁻^1^ and 5.02 t VC ha^−1^ produced yields comparable to the full inorganic rate (12.09 vs. 8.55 t ha^−1^) and achieved the highest economic return (MRR = 149%), remaining profitable under sensitivity analysis. Strong correlations between yield and soil properties (SOC, TN, available P, pH) indicate that productivity gains were directly linked to improved soil health. Among predictive models tested, the linear support vector machine (LSVM) outperformed and best fit for predicting maize grain yield under integrated nutrient management.

These results suggest that integrating BC and VC with reduced inorganic N/P_2_O_5_ can contribute to sustainable intensification by reducing dependency on costly N/P₂O₅ inputs, restoring soil fertility, and improving economic returns. However, the results showed only two cropping seasons at a single site; uniform lime application and controlled management conditions may limit generalizability. Validation through multi-location trials, longer-term studies (≥ 5 years), on-farm participatory research, assessment of biological soil quality, and evaluation of different biochar feedstocks is recommended. Subject to such validation, this integrated nutrient management approach offers a promising strategy for sustainable maize production in acidic soils of the Ethiopian highlands and similar environments.

### Study limitations and future research

This study has limitations. First, the two-year duration may not capture long-term treatment effects, particularly for biochar, which is known to exert lasting influences beyond the initial years through gradual oxidation and continued interaction with soil minerals. Second, the experiment was conducted at a single location under researcher-managed conditions; on-farm validation across multiple sites and farmer management regimes is needed to confirm these findings under real-world conditions. Third, we did not assess biological soil properties (e.g., microbial biomass, enzyme activities, earthworm populations), which could provide deeper insight into the mechanisms driving the observed yield responses. Fourth, the absence of direct pyrolysis temperature measurements, ash content, and cation exchange capacity (CEC) analysis. Fifth, the economic analysis assumed stable input and output prices, which may fluctuate in reality. Sixth, a limitation of this study is that the mineralization factor of vermicompost (0.3–0.5) was not considered prior to its application, which may have influenced nutrient availability and the observed effects on plant growth^77,94^.

### Management implications for smallholder farmers

Despite these limitations, the findings have several practical implications for farmers. Reducing inorganic fertilizer use by 50% while applying 4 t ha^−1^ of biochar and 5 t ha^−1^ of vermicompost (T14) can sustain high yields (12.09 t ha^−1^) while improving profitability (MRR = 149%), offering a viable strategy to lower cash expenditures by substituting locally produced organic amendments. Biochar can be produced from readily available crop residues, such as maize cobs and stalks, using a simple pit method during the dry season when labor is more available, thereby converting waste into a valuable soil amendment. Similarly, vermicompost production using locally available organic materials (green leaves, crop residues, and animal manure) with *Eisenia fetida* earthworms can be integrated into farming systems, contributing to both soil improvement and a potential source of income through worm sales. In addition, the split application of nitrogen fertilizer applied at planting, knee-height, and tasseling stages enhances nutrient use efficiency and should be adopted regardless of organic amendment use. Furthermore, even a moderate lime application rate (0.6 t ha^−1^), when combined with organic amendments, can effectively ameliorate soil acidity, reducing the need for the full recommended lime rate^95,96^.

### Priority areas for future research include

(i) long-term trials (≥ 10 years) to assess biochar aging, residual effects, cumulative carbon sequestration, and the sustainability of yield gains; (ii) multi-location trials across different soil types (Vertisols, Arenosols, and Andosols), agroecologies (drylands and highlands), and farming systems to establish generalizability; (iii) on-farm participatory research to evaluate performance under farmer-managed conditions, including the assessment of adoption constraints (labor, knowledge, and risk); (iv) mechanistic studies using isotopic tracers (^15^N and ^33^P) to quantify nutrient use efficiency and cycling under integrated management; (v) the inclusion of biological indicators, such as microbial biomass, community composition (phospholipid fatty acid analysis and metagenomics), enzyme activities, and soil respiration, to complement chemical measurements; (vi) optimization of biochar feedstocks and production methods to identify locally available materials and simple techniques that maximize agronomic and economic benefits; (vii) greenhouse gas measurements to assess the climate mitigation potential of biochar incorporation compared to residue burning; and (viii) modeling studies to extrapolate results across longer timeframes and broader spatial scales, thereby supporting policy decisions.

## Supplementary Information

Below is the link to the electronic supplementary material.

Supplementary Material 1

## Data Availability

The datasets analyzed during the current study are available from the corresponding author upon reasonable request.
