# Supplementary material for: Biochar–vermicompost–inorganic N/P₂O₅ integration improves maize yield and soil chemical properties in acidic soils
Source: Sci Rep. 2026 Jun 29;16:18684. doi: 10.1038/s41598-026-56112-5 (PMC13314982; doi:10.1038/s41598-026-56112-5)
Supplement: Supplementary file 1 — Supplementary Material 1 [file 41598_2026_56112_MOESM1_ESM.docx]

# **Supplementary Tables**

| Trt. | Description |
| --- | --- |
| 1 | Control (0.63 t CaCO_3_ ha⁻¹) |
| 2 | 5.02 t VC ha⁻¹ + 0.63 t CaCO_3_ ha⁻¹ |
| 3 | 10.04 t VC ha⁻¹ + 0.63 t CaCO_3_ ha⁻¹ |
| 4 | 120/69 kg N/P_2_O_5_ ha⁻¹ + 0.63 t CaCO_3_ ha⁻¹ |
| 5 | 120/69 kg N/P_2_O_5_ ha⁻¹ + 5.02 t VC ha⁻¹ + 0.63 t CaCO_3_ ha⁻¹ |
| 6 | 120/69 kg N/P_2_O_5_ ha⁻¹ ^1^ + 10.04 t VC ha⁻¹ + 0.63 t CaCO_3_ ha⁻¹ |
| 7 | 240 kg N ha⁻¹ + 138 kg P_2_O_5_ ha⁻¹ + 0.63 t CaCO_3_ ha⁻¹ |
| 8 | 240 kg N ha⁻¹ + 138 kg P_2_O_5_ ha⁻¹ + 5.02 t VC ha⁻¹ + 0.63 t CaCO_3_ ha⁻¹ |
| 9 | 240 kg N ha^-1^ + 138 kg P_2_O_5_ ha⁻¹ + 10.04 t VC ha⁻¹ + 0.63 t CaCO_3_ ha⁻¹ |
| 10 | 4 t BC ha⁻¹ + 0.63 t CaCO_3_ ha⁻¹ |
| 11 | 4 t BC ha⁻¹ + 5.02 t VC ha⁻¹ + 0.63 t CaCO_3_ ha⁻¹ |
| 12 | 4 t BC ha⁻¹ + 10.04 t VC ha⁻¹ + 0.63 t CaCO_3_ ha⁻¹ |
| 13 | 120 kg N ha⁻¹ + 69 kg P_2_O_5_ ha⁻¹ + 4 t BC ha⁻¹ + 0.63 t CaCO_3_ ha⁻¹ |
| 14 | 120 kg N ha⁻¹ + 69 kg P_2_O_5_ ha⁻¹ + 4 t BC ha⁻¹ + 5.02 t VC ha⁻¹ + 0.63 t CaCO_3_ ha⁻¹ |
| 15 | 120 kg N ha⁻¹ + 69 kg P_2_O_5_ ha⁻¹ + 4 t BC ha⁻¹ + 10.04 t VC ha⁻¹ + 0.63 t CaCO_3_ ha⁻¹ |
| 16 | 240 kg N ha⁻¹ + 138 kg P_2_O_5_ ha⁻¹ + 4 t BC ha⁻¹ + 0.63 t CaCO_3_ ha⁻¹ |
| 17 | 240 kg N ha⁻¹ + 138 kg P_2_O_5_ ha⁻¹ + 4 t BC ha⁻¹ + 5.02 t VC ha⁻¹ + 0.63 t CaCO_3_ ha⁻¹ |
| 18 | 240 kg N ha⁻¹ + 138 kg P_2_O_5_ ha⁻¹ + 4 t BC ha⁻¹ + 10.04 t VC ha⁻¹ + 0.63 t CaCO_3_ ha⁻¹ |
| 19 | 8 t BC ha⁻¹ + 0.63 t CaCO_3_ ha⁻¹ |
| 20 | 8 t BC ha⁻¹ + 5.02 t VC ha⁻¹ + 0.63 t CaCO_3_ ha⁻¹ |
| 21 | 8 t BC ha⁻¹ + 10.04 t VC ha⁻¹ + 0.63 t CaCO_3_ ha⁻¹ |
| 22 | 120 kg N ha⁻¹ + 69 kg P_2_O_5_ ha⁻¹ + 8 t BC ha⁻¹ + 0.63 t CaCO_3_ ha⁻¹ |
| 23 | 120 kg N ha⁻¹ + 69 kg P_2_O_5_ ha⁻¹ + 8 t BC ha⁻¹ + 5.02 t VC ha⁻¹ + 0.63 t CaCO_3_ ha⁻¹ |
| 24 | 120 kg N ha⁻¹ + 69 kg P_2_O_5_ ha⁻¹ + 8 t BC ha⁻¹ + 10.04 t VC ha⁻¹ + 0.63 t CaCO_3_ ha⁻¹ |
| 25 | 240 kg N ha⁻¹ + 138 kg P_2_O_5_ ha⁻¹ + 8 t BC ha⁻¹ + 0.63 t CaCO_3_ ha⁻¹ |
| 26 | 240 kg N ha⁻¹ + 138 kg P_2_O_5_ ha⁻¹ + 8 t BC ha⁻¹ + 5.02 t VC ha⁻¹ + 0.63 t CaCO_3_ ha⁻¹ |
| 27 | 240 kg N ha⁻¹ + 138 kg P_2_O_5_ ha⁻¹ + 8 t BC ha⁻¹ + 10.04 t VC ha⁻¹ + 0.63 t CaCO_3_ ha⁻¹ |

Supplementary Table S1. Treatments used in the field experiment, sowing dates and fertilizer adjustments in the 2023-24 and 2024-25 crop growing seasons. Trt, treatments; BC, biochar; VC, vermicompost. Vermicompost application rates were 0, 5.55, and 11.11 t ha⁻¹ in 2023/24, and 0, 4.48, and 8.96 t ha⁻¹ in 2024/25.

| Trt. | Three-way interaction | | | | Grain yield (t ha⁻¹) | | |
| --- | --- | --- | --- | --- | --- | --- | --- |
|  | N/P_2_O_5_  (kg ha⁻¹) | | BC  (t ha⁻¹) | VC  (t ha⁻¹) | 2023 | 2024 | Mean GY |
| T1 | 0 | | 0 | 0 | 5.13^v^ ± 0.38 | 3.67^s^ ±0.24 | 4.40^p^ ±0.19 |
| T2 | 0 | | 0 | 5.02 | 6.97^u^ ± 0.16 | 6.22^r^±0.20 | 6.59^o^ ±0.09 |
| T3 | 0 | | 0 | 10.04 | 7.38^t^ ± 0.12 | 6.95^qr^±0.17 | 7.16^n^±0.14 |
| T4 | 120/69 | | 0 | 0 | 8.17^pq^ ± 0.07 | 7.01^qr^±0.38 | 7.59^mn^±0.17 |
| T5 | 120/69 | | 0 | 5.02 | 8.37^op^ ±0.06 | 8.77^j-m^±0.07 | 8.57^j^±0.06 |
| T6 | 120/69 | | 0 | 10.04 | 8.76^mn^ ±0.08 | 8.24^m-p^±1.2 | 8.50^jk^±0.64 |
| T7 | 240/138 | | 0 | 0 | 9.36^ij^ ±0.05 | 7.74^o-q^±0.07 | 8.55^j^±0.02 |
| 8 | 240/138 | | 0 | 5.02 | 9.66^gh^ ±0.06 | 10.06^e-h^±0.06 | 9.86^gh^±0.05 |
| T9 | 240/138 | | 0 | 10.04 | 9.82^j^ ±0.05 | 10.61^c-f^±0.12 | 10.21^fg^±0.08 |
| T10 | 0 | | 4 | 0 | 7.88^rs^ ± 0.99 | 7.97^n-p^±0.08 | 7.93^lm^±0.03 |
| T11 | 0 | | 4 | 5.02 | 8.08^qr^ ± 0.03 | 8.393^l-o^±0.07 | 8.24^j-l^±0.05 |
| T12 | 0 | | 4 | 10.04 | 10.34^f^ ± 0.08 | 9.81^f-i^±0.05 | 10.07^fg^±0.06 |
| T13 | 120/69 | | 4 | 0 | 10.44^f^ ±0.12 | 10.29^d-g^±0.09 | 10.37^ef^±0.02 |
| T14 | 120/69 | | 4 | 5.02 | 11.49^c^ ±0.30 | 12.68^a^±0.93 | 12.09^a^±0.33 |
| T15 | 120/69 | | 4 | 10.04 | 11.10^d^ ±0.08 | 11.21^bc^±0.19 | 11.15^cd^±0.06 |
| T16 | 240/138 | | 4 | 0 | 11.59^c^ ±0.12 | 11.42^bc^±1.31 | 11.50^bc^±0.65 |
| T17 | 240/138 | | 4 | 5.02 | 12.19^b^ ±0.20 | 11.25^bc^±1.57 | 11.72^ab^±0.88 |
| T18 | 240/138 | | 4 | 10.04 | 10.87^e^ ±0.07 | 11.61^b^±0.91 | 11.24^c^±0.43 |
| T19 | 0 | | 8 | 0 | 7.69^s^ ±0.09 | 7.49^pq^±0.09 | 7.59^mn^±0.05 |
| T20 | 0 | | 8 | 5.02 | 7.95^r^ ±0.08 | 8.19^n-p^±0.07 | 8.07^kl^±0.04 |
| T21 | 0 | | 8 | 10.04 | 9.51^hi^ ±0.05 | 9.44^g-k^±0.06 | 9.47^hi^±0.05 |
| T22 | 120/69 | | 8 | 0 | 9.21^jk^ ± 0.05 | 9.08^i-m^±0.06 | 9.14^i^±0.03 |
| T23 | 120/69 | | 8 | 5.02 | 10.67^e^ ±0.01 | 10.88^b-e^±0.08 | 10.77^de^±0.05 |
| T24 | 120/69 | | 8 | 10.04 | 13.15^a^ ±0.01 | 11.12^b-d^±0.68 | 12.13^a^±0.34 |
| T25 | 240/138 | | 8 | 0 | 9.05^kl^ ±0.06 | 9.62^g-j^±0.07 | 9.34^i^±0.06 |
| T26 | 240/138 | | 8 | 5.02 | 8.86^lm^ ±0.12 | 9.26^h-l^±0.06 | 9.06^i^±0.03 |
| T27 | 240/138 | | 8 | 10.04 | 8.56^no^ ±0.06 | 8.58^k-o^±0.07 | 8.57^j^±0.05 |
| Mean |  | |  |  | 9.34 | 9.17 | 9.26 |
| F Test |  | |  |  | <0.0001 | 0.0001 | <0.0001 |
| LSD (0.05) |  | |  |  | 0.0698*** | 0.295*** | 0.1518*** |
| R^2^ |  | |  |  | 0.996 | 0.9525 | 0.97 |
| CV (%) |  | |  |  | 1.37 | 5.889 | 4.296 |
| Year |  | |  |  |  |  |  |
| Year 1 (2023) | |  |  |  |  |  | 9.34^a^ |
| Year 2 (2024) | |  |  |  |  |  | 9.17^b^ |
| Mean | |  |  |  |  |  | 9.258 |
| LSD (0.05) | |  |  |  |  |  | 0.5863** |
| CV (%) | |  |  |  |  |  | 20.40 |

Supplementary Table S2. Interaction effects of N/P_2_O_5_ nutrients, maize cob BC and VC on GY in 2023/24 and 2024/25 in Burie district. Trt, treatments; LSD, least significant difference; BC, biochar; CV, coefficient of variation (%); **, ***, significant at P ≤ 0.01 and P ≤ 0.001, respectively; DF, degrees of freedom. Means within a column for a given parameter followed by the same letter(s) are not significantly different at P = 0.05.

| Source | DF | Type III SS | | Mean Square | F Value | Pr > F |
| --- | --- | --- | --- | --- | --- | --- |
| Block | 2 | 0.42 | | 0.21 | 1.34 | 0.2657 |
| N/P_2_O_5_ | 2 | 189.62 | | 94.81 | 599.26 | <0.0001 |
| BC | 2 | 174.88 | | 87.44 | 552.67 | <0.0001 |
| VC | 2 | 51.81 | | 25.91 | 163.74 | <0.0001 |
| Year | 1 | 1.22 | | 1.22 | 7.70 | 0.0065 |
| N/P_2_O_5_ × BC | 4 | 55.18 | | 13.80 | 87.20 | <0.0001 |
| N/P_2_O_5_ × VC | 4 | 23.49 | | 5.87 | 37.12 | <0.0001 |
| N/P_2_O_5_ × Year | 2 | 0.82 | | 0.41 | 2.59 | 0.0801 |
| BC × VC | 4 | 6.36 | | 1.59 | 10.05 | <0.0001 |
| BC ×Year | 2 | 2.16 | | 1.08 | 6.82 | 0.0016 |
| VC × Year | 2 | 2.74 | | 1.37 | 8.65 | 0.0003 |
| N/P_2_O_5_ × BC × VC | 8 | 22.76 | | 2.85 | 17.98 | <0.0001 |
| N/P_2_O_5_ × BC × Year | 4 | 3.32 | | 0.83 | 5.25 | 0.0007 |
| N/P_2_O_5_ ×VC × Year | 4 | 4.63 | | 1.16 | 7.31 | <0.0001 |
| BC × VC × Year | 4 | 5.65 | | 1.41 | 8.93 | <0.0001 |
| N/P_2_O_5_ × BC×VC×Year | 8 | 3.64 | | 0.46 | 2.88 | 0.0061 |
| Error | 106 | 16.77 | | 0.16 |  |  |
| Corrected Total | 161 | 565.48 | |  |  |  |
| R-Square | CV | | Root MSE | Grain yield mean | |  |
| 0.970 | 4.296 | | 0.397 | 9.258 |  |  |

Supplementary Table S3. Factorial ANOVA Table on grain yield combined over year (2023-24 and 2024-25). DF, degrees of freedom; SS, sum of squares; MSE, mean square error; CV, coefficient of variation (%); GY, grain yield.

|  |  | Paired Differences | | |  | | t | df | Sig. (2-tailed) |
| --- | --- | --- | --- | --- | --- | --- | --- | --- | --- |
|  |  | Mean | Std. Deviation | Std. Error Mean | 95% Confidence Interval of the Difference | |  |  |  |
|  |  |  |  |  | Lower | Upper |  |  |  |
| Pair 1 | pre_pH - pos_pH | -0.448 | 0.275 | 0.031 | -0.508 | -0.387 | -14.680 | 80 | < 0.001 |
| Pair 2 | Pre_TN - pos_TN | -0.036 | 0.028 | 0.003 | -0.042 | -0.030 | -11.482 | 80 | < 0.001 |
| Pair 3 | pre_avP - pos_avP | -20.669 | 7.832 | 0.870 | -22.401 | -18.938 | -23.753 | 80 | < 0.001 |
| Pair 4 | pre_SOC - pos_SOC | -2.315 | 0.209 | 0.023 | -2.361 | -2.269 | -99.478 | 80 | < 0.001 |
| Pair 5 | pre_SOM - pos_SOM | -0.770 | 0.365 | 0.041 | -0.851 | -0.690 | -19.005 | 80 | < 0.001 |
| Pair 6 | pre_Exc. H - pos_Exc. H | 0.525 | 0.174 | 0.019 | 0.487 | 0.564 | 27.158 | 80 | < 0.001 |
| Pair 7 | pre_Exc. Al - pos_Exc. Al | 0.265 | 0.266 | 0.030 | 0.206 | 0.324 | 8.965 | 80 | < 0.001 |
| Pair 8 | pre_Exc. Ac - pos_Exc. Ac | 0.790 | 0.423 | 0.047 | 0.697 | 0.884 | 16.810 | 80 | < 0.001 |
| Pair 9 | Yield_1_ – Yield_2_ | 0.173 | 0.889 | 0.099 | -0.023 | 0.370 | 1.754 | 80 | 0.083 |

Supplementary Table S4. Paired-samples test comparing soil chemical properties and grain yield before the experiment and after two years. df, degrees of freedom; pre, before the experiment; pos, after the experiment (two years later); t, test statistic used to determine if the mean difference is statistically significant. pH, soil reaction; TN, total nitrogen; avP, available phosphorus; SOC, soil organic carbon; SOM, soil organic matter; Exc. H, Exc. Al, and Exc. Ac, exchangeable hydrogen, aluminum, and acidity; Yield_1_, maize yield (2023/24); Yield_2_, maize yield (2024/25). Number of observations = 81.
